# Design, synthesis, and inhibitory activity of hydroquinone ester derivatives against mushroom tyrosinase†

**DOI:** 10.1039/d4ra00007b

**Published:** 2024-02-16

**Authors:** Dong Xie, Kangjia Han, Qian Jiang, Sida Xie, Jielong Zhou, Yingjun Zhang, Junming Xu, Yuanping He, Ping Zhao, Xiaoqin Yang

**Affiliations:** a Key Laboratory of National Forestry and Grassland Administration on Highly-Efficient Utilization of Forestry Biomass Resources in Southwest China, Southwest Forestry University Kunming Yunnan 650224 PR China yangxiaoqin@swfu.edu.cn; b Laboratory of Phytochemistry and Plant Resources in West China, Kunming Institute of Botany, Chinese Academy of Sciences Kunming Yunnan 650204 PR China; c Institute of Chemical Industry of Forest Products, Chinese Academy of Forestry Nanjing Jiangsu 210042 PR China; d Kunming Beiye Dai Medicine Research Institute Kunming Yunnan 650499 PR China; e Key Laboratory of Ministry of Education for Forest Resources Conservation and Utilization in the Southwest Mountains of China, Southwest Forestry University Kunming Yunnan 650224 PR China hypzhao2022@163.com

## Abstract

Tyrosinase is a widely distributed copper-containing enzyme found in various organisms, playing a crucial role in the process of melanin production. Inhibiting its activity can reduce skin pigmentation. Hydroquinone is an efficient inhibitor of tyrosinase, but its safety has been a subject of debate. In this research, a scaffold hybridization strategy was employed to synthesize a series of hydroquinone–benzoyl ester analogs (3a–3g). The synthesized compounds were evaluated for their inhibitory activity against mushroom tyrosinase (mTyr). The results revealed that these hydroquinone–benzoyl ester analogs exhibited inhibitory activity against mTyr, with compounds 3a–3e displaying higher activity, with compound 3b demonstrating the highest potency (IC_50_ = 0.18 ± 0.06 μM). Kinetic studies demonstrated that the inhibition of mTyr by compounds 3a–3e was reversible, although their inhibition mechanisms varied. Compounds 3a and 3c exhibited non-competitive inhibition, while 3b displayed mixed inhibition, and 3d and 3e showed competitive inhibition. UV spectroscopy analysis indicated that none of these compounds chelated with copper ions in the active center of the enzyme. Molecular docking simulations and molecular dynamics studies revealed that compounds 3a–3e could access the active pocket of mTyr and interact with amino acid residues in the active site. These interactions influenced the conformational flexibility of the receptor protein, subsequently affecting substrate–enzyme binding and reducing enzyme catalytic activity, in line with experimental findings. Furthermore, *in vitro* melanoma cytotoxicity assay of compound 3b demonstrated its higher toxicity to A375 cells, while displaying low toxicity to HaCaT cells, with a dose-dependent effect. These results provide a theoretical foundation and practical basis for the development of novel tyrosinase inhibitors.

## Abstract 2

Tyrosinase is a widely distributed copper-containing enzyme found in various organisms, playing a crucial role in the process of melanin production.

## Graphical abstract

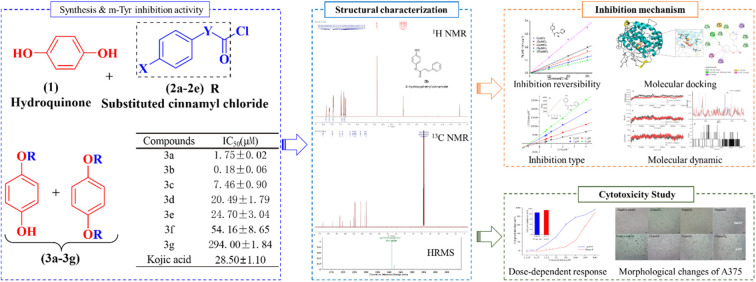

## Introduction

Tyrosinase (EC 1.14.18.1) is a crucial and rate-limiting enzyme responsible for catalyzing melanin synthesis. It is widely distributed in living organisms and intricately linked to numerous pivotal physiological processes. Tyrosinase possesses a complex structure with an active site containing a binuclear copper center situated within the active region. In the presence of oxygen, it efficiently catalyzes the oxidation of phenolic compounds.^1^ In mammals, tyrosinase is predominantly located in melanocytes, playing a pivotal role throughout the melanin formation process. Disorders in tyrosinase metabolism within the human body can result in pigmentation-related skin conditions such as melasma, freckles, and age spots. Moreover, these metabolic irregularities are directly associated to the onset and treatment of various human diseases, including Parkinson's disease, melanoma, and albinism.^2^ Research has indicated that inhibiting tyrosinase activity to impede melanin production is an effective approach for treating pigmentation-related skin disorders.^3^ Hydroquinone, a potent tyrosinase inhibitor, was first proposed by Oettel^4^ in 1936 for its skin-whitening effects. By the 1960s, it commenced utilization in cosmetics as a depigmenting agent and in topical formulations for treating pigmentation disorders in dermatology. Nevertheless, it was discovered that hydroquinone could be skin-irritating, and prolonged use could lead to a severe condition known as exogenous ochronosis.^5^ Consequently, the inclusion of hydroquinone in skincare products has been prohibited in China and Europe, though it remains permitted for pharmaceutical use.

**Fig. 1:**
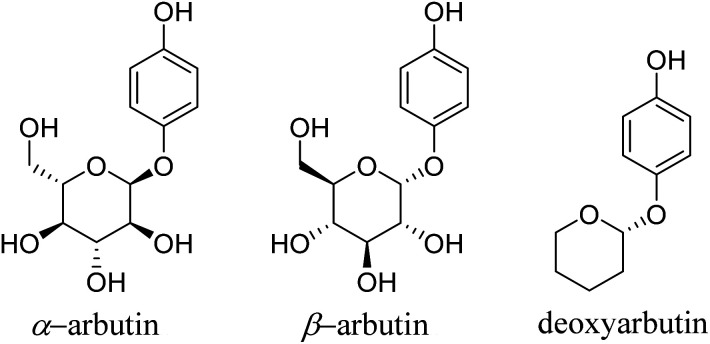
Chemical structure of arbutin derivates.

In 1996, Maeda^6^ discovered that arbutin, a glycosylated derivative of hydroquinone (Fig. 1), exhibited skin-whitening and depigmenting effects, effectively treating conditions like melasma and melanoma.^7^ Shiseido in Japan was among the pioneering companies to incorporate it as a natural additive in cosmetics, asserting it to be a non-toxic, side-effect-free substance.

The Personal Care Products Council in the United States included “arbutin extract” in the U.S. Cosmetic Ingredient Dictionary, and China also listed arbutin in the Catalog of Cosmetic Raw Materials (2015 Edition).^8^ However, recent years have seen safety concerns and adverse effects related to arbutin emerging within the cosmetic industry, prompting many companies to seek safer and more effective alternatives. Structural modifications and hybrid scaffold strategies represent vital approaches for expanding the biological activities of scaffolds and discovering highly active molecules. Hydroquinone esters commonly serve as synthetic intermediates for drugs.^9^ Due to their unique chemical structure, they can be modified to fine-tune the activity and bioavailability of drugs. Benzoyl compounds comprise a range of organic compounds with the benzoyl (C_6_H_5_CO–) functional group, exhibiting diverse biological activities such as antimicrobial, anti-inflammatory, antioxidant, anticancer, analgesic, and insecticidal effects.^10,11^ Specific benzoyl compounds, such as cinnamic acid derivatives, also demonstrate a degree of inhibition on tyrosinase, affecting the skin pigmentation process.^12^ Hence, the synthesis of hydroquinone with benzoyl compounds, using a scaffold hybridization strategy, offers promise for novel, highly efficient, and low-toxicity tyrosinase inhibitors.

Among various tyrosinase, mushroom tyrosinase (mTyr) is routinely used in experiments due to its well-established laboratory preparation methods.^13^ Therefore, in this research, hydroquinone and benzoyl derivatives were hybridized to synthesize a series of hydroquinone–benzoyl ester analogs. These synthesized compounds were evaluated for their inhibitory activity against mTyr, and their inhibitory activity against melanoma. The goal is to discover potent and safe tyrosinase inhibitors, holding significance in various fields, including food and pharmaceuticals.

## Results and discussion

### Synthesis of hydroquinone ester derivatives

**Scheme 1:**
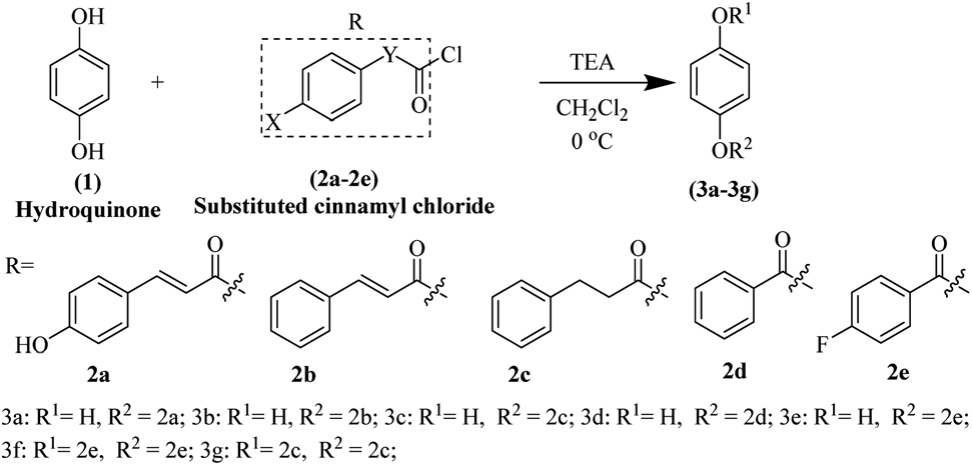
Synthesis of hydroquinone ester derivatives 3a–3g.

The hydroquinone ester derivatives were prepared using the acylation between the hydroquinone (1) and different substituted benzoyl chloride compounds 2a–2e.^14^ Compounds 3a–3g were obtained in satisfactory yields (40–75%) after purification by silica gel column chromatography (Scheme 1). The reaction formed a mixture of mono-substituted and di-substituted compounds from which the purification of 3c by silica gel column chromatography was possible.

The synthesis route of hydroquinone ester derivatives is depicted in Scheme 1. The reaction of hydroquinone with substituted benzoyl chloride yielded the target compounds 3a–3g (Scheme 1), among which 3a is novel compound, while 3b–3g are novel compounds. All the compounds were characterized using NMR (^1^H and ^13^C) and HRMS, and spectroscopic data can be found in the ESI (Fig. S1 and S2†).

Compound 3a, 4-hydroxyphenyl (2*E*)-3-(4-hydroxyphenyl) prop-2-enoate, pale yellow solid, obtained in 45% yield, m.p. 217–220 °C, purified by silica gel column chromatography eluted with petroleum ether/ethyl acetate (2 : 1, v/v), TLC: *R*_f_ = 0.25. UV-Vis (200–450 nm, CH_3_OH) *λ*: 314 nm. ^1^H NMR (500 MHz, CD_3_OD) *δ* 7.76 (d, 1H, *J* = 15.9 Hz), 7.53–7.51 (m, 2H), 6.95–6.93 (m, 2H), 6.84–6.77 (m, 5H), 6.61 (s, 1H), 6.50 (d, 1H, *J* = 16.0 Hz). ^13^C NMR (126 MHz, CD_3_OD) *δ*: 168.41, 161.78, 156.44, 148.15, 145.07, 131.63, 127.22, 123.62, 117.07, 116.78, 114.64. HRMS (ESI, 70 eV) calcd for C_15_H_12_O_4_ [M + H]^+^, *m*/*z* 256.0736; found, *m*/*z* 257.0808.

Compound 3b, 4-hydroxyphenyl cinnamate, white solid, obtained in 75% yield, m.p. 171–173 °C, purified by silica gel column chromatography eluted with petroleum ether/ethyl acetate (2 : 1, v/v), TLC: *R*_f_ = 0.25. UV-Vis (200–450 nm, CH_3_OH) *λ*: 283 nm. ^1^H NMR (500 MHz, CD_3_OD) *δ* 7.84 (d, 1H, *J* = 16.0 Hz), 7.68–7.64 (m, 2H), 7.46–7.40 (m, 3H), 7.00–6.93 (m, 2H), 6.82–6.78 (m, 2H), 6.71 (d, *J* = 16.0 Hz, 1H). ^13^C NMR (126 MHz, CD_3_OD) *δ* 166.19, 154.98, 146.27, 143.42, 134.24, 130.42, 128.70, 128.05, 121.99, 116.93, 115.25. HRMS (ESI, 70 eV) calcd for C_15_H_12_O_3_ [M + H]^+^, *m*/*z* 241.0786; found, *m*/*z* 241.0859.

Compound 3c, 4-hydroxyphenyl 3-phenylpropanoate, white solid, obtained in 40% yield, m.p. 149–151 °C, purified by silica gel column chromatography eluted with petroleum ether/ethyl acetate (7 : 1, v/v), TLC: *R*_f_ = 0.25. UV-Vis (200–450 nm, CH_3_OH) *λ*: 273 nm. ^1^H NMR (500 MHz, CDCl_3_) *δ* 7.37–7.21 (m, 5H), 6.87–6.81 (m, 2H), 6.78–6.71 (m, 2H), 5.16 (d, 1H, *J* = 15.4 Hz), 3.12–2.81 (m, 4H). ^13^C NMR (126 MHz, CDCl_3_) *δ* 172.18, 153.34, 144.03, 140.08, 128.60, 128.40, 126.46, 122.39, 115.98, 35.95, 30.96. HRMS (ESI, 70 eV) calcd for C_15_H_14_O_3_ [M + H]^+^, *m*/*z* 243.0943; found, *m*/*z* 243.1016.

Compound 3d, 4-hydroxyphenyl benzoate, white solid, obtained in 65% yield, m.p. 150–152 °C, purified by silica gel column chromatography eluted with petroleum ether/ethyl acetate (7 : 1, v/v), TLC: *R*_f_ = 0.25. UV-Vis (200–450 nm, CH_3_OH) *λ*: 272 nm, 314 nm. ^1^H NMR (500 MHz, CD_3_OD) *δ* 8.15 (dt, 2H, *J* = 8.5, 1.5 Hz), 7.67 (tdd, 1H, *J* = 7.1, 2.3, 1.2 Hz), 7.58–7.50 (m, 2H), 7.05–6.99 (m, 2H), 6.86–6.79 (m, 2H). ^13^C NMR (126 MHz, CD_3_OD) *δ*: 167.17, 156.50, 144.91, 134.76, 130.98, 130.96, 129.77, 123.46, 116.73. HRMS (ESI, 70 eV) calcd for C_13_H_10_O_3_ [M + H]^+^, *m*/*z* 215.0630; found, *m*/*z* 215.0703.

Compound 3e, 4-hydroxyphenyl 4-fluorobenzoate, white solid, obtained in 55% yield, m.p. 142–144 °C, purified by silica gel column chromatography eluted with petroleum ether/ethyl acetate (7 : 1, v/v), TLC: *R*_f_ = 0.25. UV-Vis (200–450 nm, CH_3_OH) *λ*: 272 nm. ^1^H NMR (500 MHz, CDCl_3_) *δ* 8.21 (dd, 2H, *J* = 8.7, 5.5 Hz), 7.18 (t, 2H, *J* = 8.6 Hz), 7.04–7.00 (m, 2H), 6.82–6.78 (m, 2H), 5.56 (s, 1H). ^13^C NMR (126 MHz, CDCl_3_) *δ* 167.23, 165.20, 165.12, 153.64, 144.09, 132.87, 132.79, 125.69, 125.67, 122.49, 116.21, 115.92, 115.74. HRMS (ESI, 70 eV) calcd for C_13_H_9_FO_3_ [M + H]^+^, *m*/*z* 233.0536; found, *m*/*z* 233.0608.

Compound 3f, 1,4-phenylene bis(4-fluorobenzoate), white solid, obtained in 40% yield, m.p. 196–198 °C, purified by silica gel column chromatography eluted with petroleum ether/ethyl acetate (15 : 1, v/v), TLC: *R*_f_ = 0.25. UV-Vis (200–450 nm, CH_3_OH) *λ*: 241 nm. ^1^H NMR (500 MHz, CDCl_3_) *δ* 8.27–8.20 (m, 4H), 7.28 (s, 4H), 7.20 (t, *J* = 8.6 Hz, 4H). ^13^C NMR (126 MHz, CDCl_3_) *δ* 167.26, 165.23, 164.11, 148.34, 132.89, 132.82, 125.59, 125.57, 122.67, 115.96, 115.78, 77.27, 77.02, 76.76. HRMS (ESI, 70 eV) calcd for C_20_H_12_F_2_O_4_ [M + H]^+^, *m*/*z* 355.0704; found, *m*/*z* 355.0777.

Compound 3g, 1,4-phenylene bis(3-phenylpropanoate), white solid, obtained in 57% yield, m.p. 128–130 °C, purified by silica gel column chromatography eluted with petroleum ether/ethyl acetate (15 : 1, v/v), TLC: *R*_f_ = 0.25. UV-Vis (200–450 nm, CH_3_OH) *λ*: 228 nm, 260 nm. ^1^H NMR (500 MHz, chloroform-*d*) *δ* 7.35–7.28 (m, 4H), 7.28–7.20 (m, 6H), 6.99 (s, 4H), 3.06 (t, 4H, *J* = 7.7 Hz), 2.88 (dd, 4H, *J* = 8.1, 7.2 Hz). ^13^C NMR (126 MHz, CDCl_3_) *δ* 171.26, 148.00, 140.03, 128.61, 128.40, 126.48, 122.36, 35.93, 30.92. HRMS (ESI, 70 eV) calcd for C_24_H_22_O_4_ [M + H]^+^, *m*/*z* 375.1518; found, *m*/*z* 375.1592.

### Inhibitory activity of compounds 3a–3g on mTyr

Compounds 3a to 3g were subjected to evaluation for their inhibitory activity against mTyr using l-tyrosine as the substrate. The data presented in Table 1 and Fig. S3† reveal that all these compounds demonstrated significant inhibitory effects on mTyr, with maximum inhibition rates exceeding 50%. Notably, compound 3b displayed the highest inhibition rate, reaching (80.56 ± 2.48)%. Subsequently, the IC_50_ values of the compounds were determined. In comparison, the parent compounds hydroquinone and 4-hydroxycinnamic acid exhibited strong inhibitory activities, with IC_50_ values of (22.78 ± 0.16) and (16.45 ± 5.42) μM, respectively, while cinnamic acid displayed a more moderate mTyr inhibition with an IC_50_ greater than 200 μM. Interestingly, the esterified products 3a to 3e demonstrated even higher inhibitory activities, exhibiting IC_50_ values ranging from (0.18 ± 0.06) to (24.70 ± 3.04) μM. This indicated a significant enhancement in inhibition compared to the parent compounds and the positive control kojic acid [IC_50_ = (28.50 ± 1.10) μM].^15^ The results suggest that the hybridization of hydroquinone and substituted benzoyl led to improved bioactivity of the scaffold, resulting in better binding with mTyr and yielding compounds with higher mTyr inhibitory activity. These findings hold valuable implications for the design and development of related mTyr inhibitors.

**Table 1:** IC_50_ values of compounds 3a–3g for inhibiting mTyr activity

Compounds	IC_50_ (μM)	Maximum inhibition rate/concentration (%/μM)
3a	1.75 ± 0.02	74.66 ± 2.44/10
3b	0.18 ± 0.06	80.56 ± 2.48/2.5
3c	7.46 ± 0.90	75.41 ± 1.41/50
3d	20.49 ± 1.79	68.74 ± 1.97/75
3e	24.70 ± 3.04	68.27 ± 2.83/100
3f	54.16 ± 8.65	69.50 ± 0.70/100
3g	294.00 ± 1.84	50.00 ± 1.41/250
Hydroquinone	22.78 ± 0.16	78.50 ± 3.54/75
Cinnamic acid	201.40 ± 5.30 (ref. 15)	—
4-Hydroxycinnamic acid	16.45 ± 5.42	71.82 ± 7.76/50
Kojic acid	28.50 ± 1.10 (ref. 15)	—

Moreover, according to the results of their inhibitory mTyr activity, five compounds 3a–3e with IC_50_ value lower than kojic acid [IC_50_ = (28.50 ± 1.10) μM] were selected for further mechanism study.

### Inhibition reversibility and type of compounds 3a–3e on mTyr

**Fig. 2:**
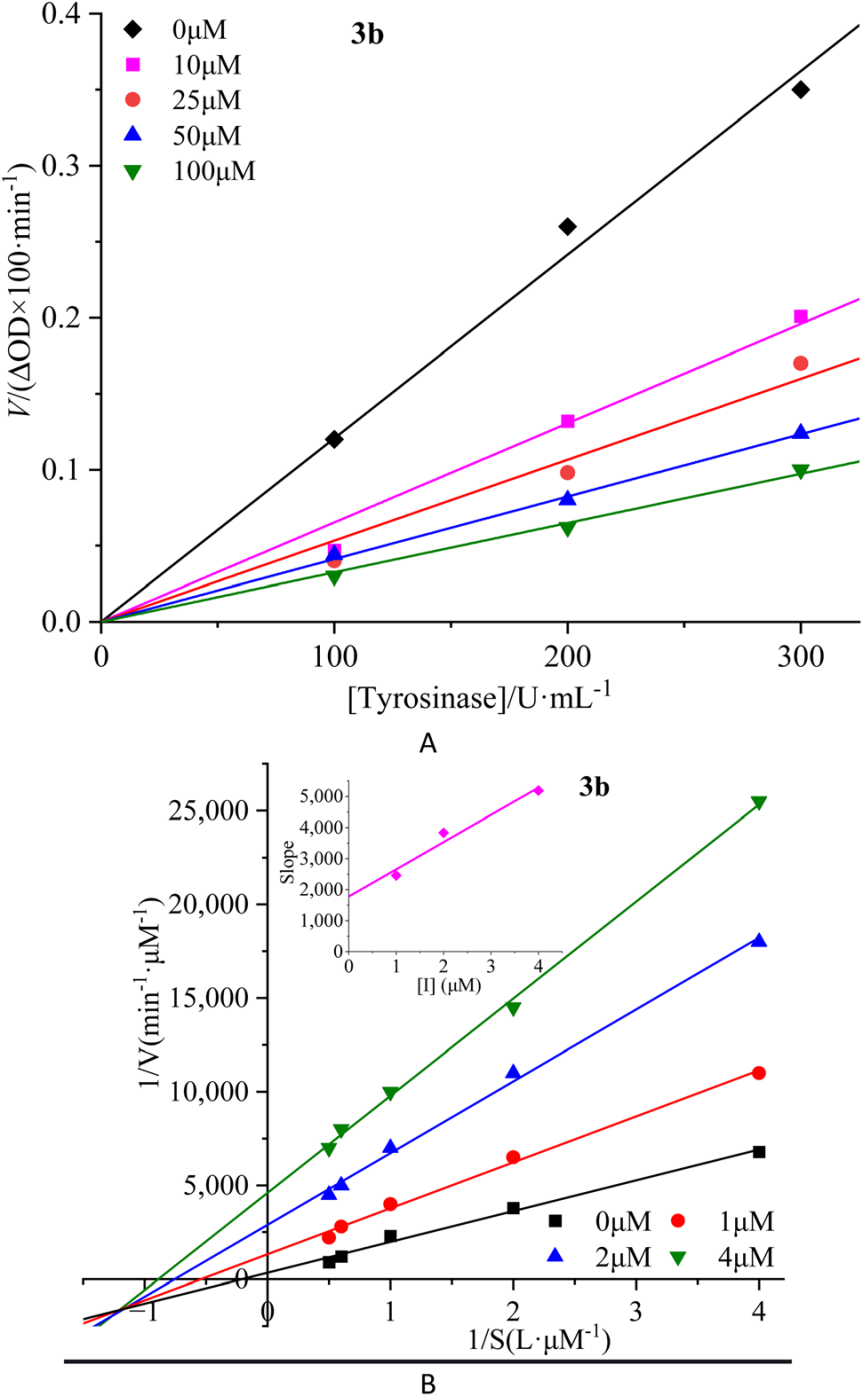
Inhibition reversibility (A) and inhibition type (B) of compound 3b on mTyr. The embed (B) in the left represent the secondary slope of the straight lines *versus* concentration of compound 3b.

To investigate the reversibility of compounds inhibition on mTyr, a dynamic system was employed. The concentration of substrate l-tyrosine remained constant while varying the concentration of mTyr. Different concentrations of compounds were tested to evaluate their impact on the initial catalytic rate of enzyme under inhibitory conditions. By analyzing the relationship between the initial reaction rate of the enzyme-catalyzed reaction and the enzyme concentration for different compound concentrations, the reversibility of the inhibition process could be determined. The outcomes revealed distinct linear relationships originating from the origin for each compound (3a–3e) upon interaction with mTyr (Fig. 2A for 3b and S4A† for 3a, 3c–3e).

Notably, as the concentration of the compounds increased, the slope of these lines gradually decreased. This trend indicates that the inhibition exerted by these compounds on mTyr activity is reversible. Furthermore, the rise in compound concentration led to a corresponding decline in enzyme activity. This observation suggests that these compounds engage in a reversible binding process with mTyr, forming dissociable complexes that impede the catalytic activity of enzyme. This reversible binding does not seem to induce permanent changes in the molecular conformation of enzyme that would lead to inactivation. Further probing the inhibition types of compounds on mTyr, a constant enzyme concentration was maintained while altering the substrate concentration of l-tyrosine. The impact of various compound concentrations on the enzyme-catalyzed reaction rate was determined within this experimental system. Utilizing the Lineweaver–Burk equation, double reciprocal plots were constructed, and the intersection points of the lines were employed to discern the inhibition types induced by the compounds (Fig. 2B for 3b and S4B† for 3a, 3c–3e). Notably, each compound (3a–3e) yielded a set of well-fitted linear relationships.^16^

However, distinct compounds exhibited intersecting lines in different quadrants, signifying diverse inhibition types. From Fig. S4B† for compound 3a and 3c, it is evident that upon introducing varying concentrations of 3a and 3c, the derived lines intersected the negative *X*-axis. Calculating the slope and intercept of these lines provided the Michaelis constant (*K*_m_) and maximum reaction rate (*V*_m_) of mTyr, respectively.^15^ Table S1† revealed that while *K*_m_ remained constant with altering 3a and 3c concentrations, *V*_m_ decreased with escalating compound concentrations. This indicated that compounds 3a and 3c function as non-competitive inhibitors of mTyr.^17^ This observation suggests that these compounds engage with essential groups (such as catalytic moieties) other than the substrate binding site in the active centre of enzyme. Consequently, the inhibitors do not diminish the affinity of enzyme for the substrate, but rather impede its catalytic function, thus reducing *V*_m_. Additionally, within this inhibition type, compounds 3a and 3c can form a ternary enzyme–compound–substrate complex.^15^ Although this binding mode does not influence the enzyme–substrate interaction, it obstructs further product formation and consequently leads to diminished enzyme activity.^18^

For compound 3b (Fig. 2B), the lines intersect the third quadrant. Upon introducing different concentrations of 3b, the determined *K*_m_ and *V*_m_ values were observed to change, with *K*_m_ decreasing and *V*_m_ increasing with higher 3b concentrations (Table S1†). This denotes a mixed-type inhibition for compound 3b, indicating its interaction with both free mTyr and the mTyr–substrate complex. As shown in Fig. 2B, creating secondary plots of 1/*V*_m_ and *K*_m_ for various 3b concentrations facilitated the calculation of the free enzyme inhibition constant (*K*_I_) and the mTyr–substrate complex inhibition constant (*K*_IS_), which were found to be 1.8831 μM and 0.3395 μM, respectively. Notably, *K*_IS_ was lower than *K*_I_, suggesting that compound 3b has a propensity to associate with the enzyme–substrate complex.^19^

For compounds 3d and 3e (Fig. S4B†), the lines intersected the *Y*-axis. The consistent *V*_m_ values, despite changing 3d and 3e concentrations, indicated that these compounds solely influence *K*_m_. The increase in *K*_m_ with rising 3d and 3e concentrations indicates their role as competitive inhibitors of mTyr (Table S1†). The interaction of 3d and 3e with free mTyr impedes the substrate l-tyrosine from binding to the active site of enzyme. As a result, 3d and 3e competitively bind to free mTyr, hindering the binding of the substrate l-tyrosine. This mutual exclusion between the substrate and inhibitors reduces enzyme activity.

In conclusion, this study uncovers the distinct inhibition types exhibited by compounds 3a–3e based on their interaction with mTyr, copper ions, and substrates. These findings provide crucial insights into the molecular mechanisms underlying the inhibitory effects of these compounds.

### Ability of compounds to chelate copper(ii) ions

**Fig. 3:**
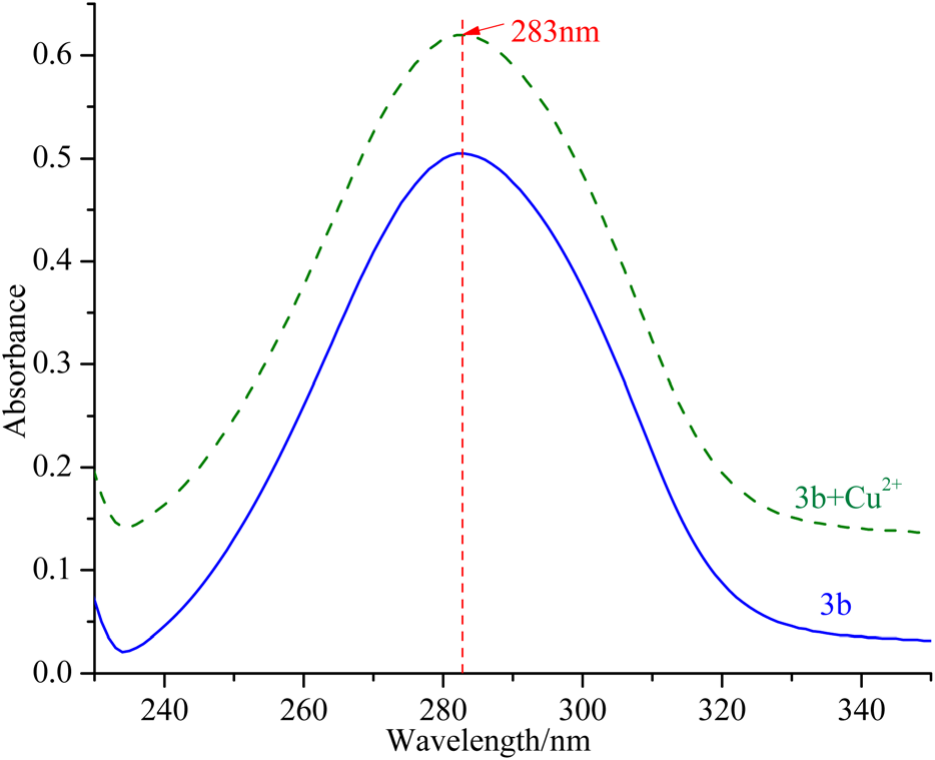
UV spectra of compound 3b before and after interaction with copper(ii) ions.

mTyr is a metalloenzyme, and the bimetallic copper(ii) ions within its active site play a crucial role. To investigate whether compounds 3a–3e can chelate with the copper(ii) ions within the active site of mTyr, we employed UV-Visible spectroscopy.^20^ Experimental results, as depicted in the Fig. 3 and S5,† reveal that when a certain concentration of copper(ii) ions is introduced into the solution of compounds 3a–3e, there is no shift observed in the UV absorption peaks of compounds 3a–3e. This phenomenon indicates that when copper(ii) ions are added to the solution of compounds 3a–3e, no chelation occurs between them. This suggests that the inhibitory activity exhibited by these compounds on mTyr may be due to the formation of new complexes with amino acid residues within the active site of mTyr.

### Molecular stimulation of compounds 3a–3e with mTyr

#### Molecular docking

**Fig. 4:**
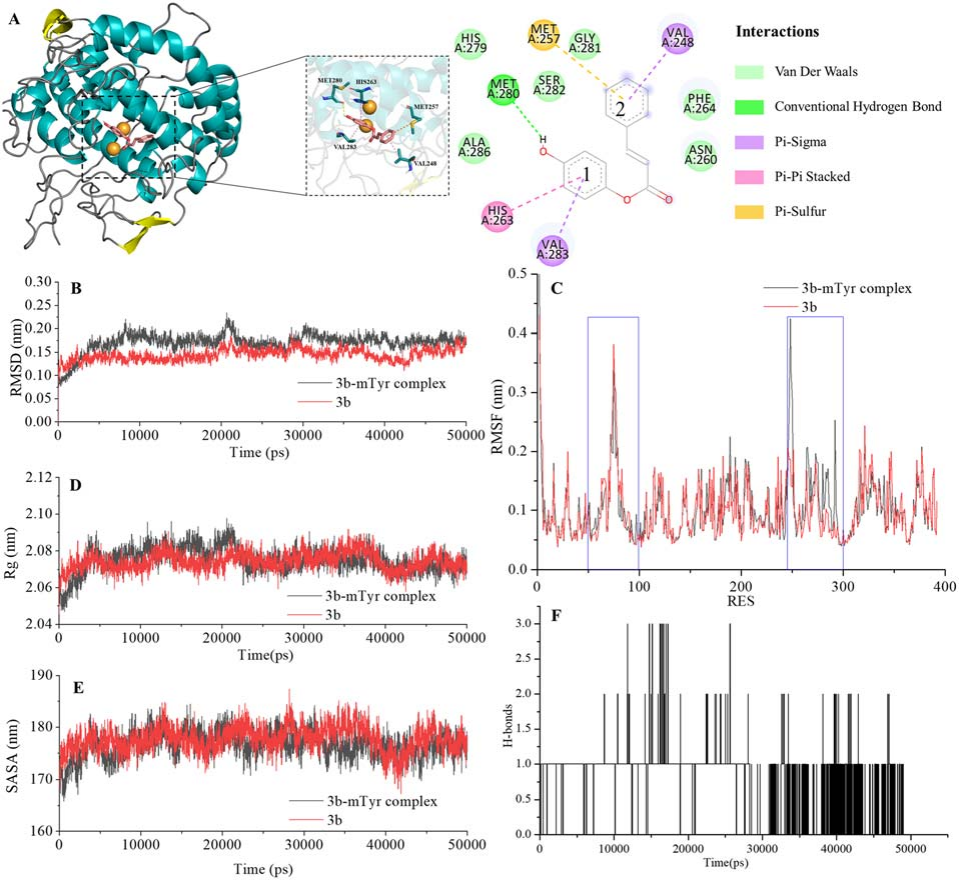
Docking model for compound 3b with mTyr (A) and molecular dynamics results of compound 3b and 3b–mTyr complex with: (B) RMSD, (C) RMSF, (D) *R*_g_, (E) SASA, and (F) H-bonds.

To gain deeper insights into the interaction mechanisms between compounds 3a–3e and mTyr, we conducted molecular docking studies of each of these five compounds with mTyr. The crystal structure of mTyr (PDB ID: 2Y9X)^21^ comprises four structurally similar domains (Fig. S6†), each hosting an active site containing two copper ions and specific amino acids. Therefore, any of these domains can be chosen for molecular docking analysis. For this study, we selected domain A for molecular docking analysis and selected the binding conformation with the lowest energy binding from 20 docking runs as the optimal binding mode of compounds 3a–3e with mTyr, as illustrated in the Fig. 4A and S7A,† and summarized in the Table S2.†

From the figures, it is clear that although each compound adopts a distinct binding conformation with mTyr, compounds 3a–3e can access the active pocket of mTyr effectively. They approach the active centre copper ions and interact with both hydrophilic and hydrophobic amino acid residues surrounding the active centre through various forces, including van der Waals forces, hydrogen bonds (conventional hydrogen bonds and carbon hydrogen bonds), and hydrophobic interactions (pi–pi stacked/T-shaped, pi–sigma, and pi–alkyl interactions). These findings suggest that compounds 3a–3e do not directly interact with the copper ions in the active centre of mTyr, which aligns with the results of the UV-Visible spectroscopy analysis as mentioned earlier. As shown in Table S2,† the docking energies for compounds 3a–3e are −7.6, −7.2, −6.7, −7.0, and −6.7 kcal mol^−1^, respectively. Typically, the presence of hydrogen bonds enhances the strength of non-covalent interactions between small molecules and proteins. In the case of compounds 3a–3e, the phenolic hydroxyl group provided by the hydroquinone moiety in the compound structure can form hydrogen bonds with some amino acid residues. Specifically, in compound 3a, the phenolic hydroxyl group forms a conventional hydrogen bond with Arg268 with a bond length of 6.66 Å. In compound 3b, the phenolic hydroxyl group forms a conventional hydrogen bond with Met280 with a bond length of 5.01 Å. In compound 3d, the phenolic hydroxyl group forms conventional hydrogen bonds with Met280 and His263 with bond lengths of 4.88 Å and 4.06 Å, respectively. On the other hand, the phenolic hydroxyl groups in compounds 3c and 3e do not form hydrogen bonds with amino acid residues. Instead, the non-hydroquinone moiety ends of compound 3c engage in additional interaction forces with amino acids surrounding the active centre. The benzene ring in the non-hydroquinone moiety part of compound 3c engages in pi–sigma hydrophobic stacking interactions with Val283, pi–pi stacked/T-shaped interactions with His85 and His263. In the field of drug design, fluorine is usually introduced into small molecule compounds to inhibit sites prone to oxidation metabolism. This enhances the metabolic stability of the compounds and extends their duration of action within the body.^22^ In compound 3e, the fluorine atom also engages in halogen interactions with His263, which bears structural significance similar to weak hydrogen bonding. Additionally, the benzene ring connected to the fluorine in 3e forms pi–alkyl conjugation with Ala286 and Val283, pi–pi stacked/T-shaped hydrophobic interactions with His 85 and His263. It is worth noting that in compounds 3a, 3b, and 3c, the benzene rings within the hydroquinone moiety interact with Met257 through pi–sulfur interactions. In summary, compounds 3a, 3b, and 3d all exhibit docking energies below −7 kcal mol^−1^, indicating a strong binding affinity with mTyr, whereas compounds 3c and 3e exhibit docking energies within the range of −4 to −7 kcal mol^−1^, signifying moderate binding strengths. These results highlight the importance of hydrogen bonds and pi–sulfur interactions in the process of compound binding to mTyr. These non-covalent interactions induce modifications in the conformation of the mTyr active centre and, to varying degrees, impact the microenvironment of amino acid residues, consequently influencing substrate–enzyme binding. Taking into consideration the IC_50_ values of compounds against mTyr and the structural analysis, it becomes clear that the incorporation of double bonds conjugated with benzene rings enhances the formation of robust hydrogen bond interactions between the compounds and mTyr. Additionally, the introduction of fluorine atoms elevates the hydrophobicity characteristics of the molecules, thereby enhancing their activity. These findings offer valuable insights for the design and synthesis of highly potent mTyr inhibitors.^23^

#### Molecular dynamics

Molecular docking provides insights into the binding sites and modes of interaction between small molecule ligands and mTyr enzyme receptors. However, it cannot describe the dynamic evolution of their interactions or the stability of the complex system. Therefore, the use of molecular dynamics simulations to study the compound–mTyr complex system can enhance our understanding of their interaction dynamics.^23^

The Root Mean Square Deviation (RMSD) of main chain carbon atoms is often employed to evaluate the stability and accuracy of docking results.^24^ The molecular dynamics trajectory results, as shown in the Fig. 4B and S7B,† indicate that the RMSD values of the empty mTyr protein receptor undergo significant fluctuations within the initial 10 000 ps but reach equilibrium after 30 000 ps, stabilizing at around 0.17 nm. This suggests that the structure of the mTyr protein remains stable during the molecular dynamics process. For the compound (3a–3e)–mTyr complex systems, the RMSD values tend to stabilize in the range of 35 000–45 000 ps, indicating reliable dynamic equilibrium in the compound (3a–3e)–mTyr complex systems. This provides strong evidence for the stable existence of the compound (3a–3e)–mTyr complexes. Notably, the RMSD values for the 3a–mTyr and 3b–mTyr complex systems are lower than those for the empty mTyr protein receptor, indicating lower mobility and better stability of the complex systems. This trend aligns with the inhibitory capacity of the five compounds on mTyr, with experimental results showing that compounds 3a and 3b exhibit better activity.

The Root Mean Square Fluctuation (RMSF) values of amino acid residues provide insights into the flexibility and local motion characteristics of the system. As shown in the Fig. 4C and S7C,† amino acid residues in the compound (3a–3e)–mTyr complex systems exhibit pronounced fluctuations in two regions, namely, 50–100 and 240–300. This indicates that small molecule compounds interact with nearby amino acid residues within the active pocket and that these amino acid residues actively participate in and stabilize the binding process by adjusting their conformations and angles.^25^

The radius of gyration (*R*_g_) can characterize the structural compactness and flexibility of protein molecules. A larger *R*_g_ value indicates greater structural relaxation. The Fig. 4D and S7D† illustrates that the empty mTyr protein receptor stabilizes at an *R*_g_ value of around 2.07 nm after 20 000 ps. Similarly, the compound (3a–3e)–mTyr complex systems also stabilize after 20 000 ps, with *R*_g_ values slightly higher than those of the empty mTyr protein receptor. Amino acid residues repel each other due to interactions, leading to protein structural relaxation and expansion. This suggests that as small molecule compounds penetrate deeper into the active pocket and interact with surrounding amino acid residues, these residues undergo conformational changes, causing the protein structure to relax and the *R*_g_ to increase.^26^

The Solvent Accessible Surface Area (SASA) reflects the area of the surface of protein receptor surface in contact with solvent molecules and can characterize its hydrophobicity. As shown in Fig. 4E and S7E,† the SASA of the empty mTyr protein receptor remains stable at approximately 178 nm throughout the simulation process. In contrast, the compound–mTyr complex systems exhibit more noticeable variations, ultimately resulting in slightly higher SASA values than the empty mTyr protein receptor. This indicates that during the simulation process, as the protein becomes more relaxed, the surface area accessible to solvent molecules increases. This result is consistent with the *R*_g_ analysis.^27^

Hydrogen bonds play a significant role in substrate recognition and maintaining the stability of small molecule ligand–protein receptor complexes.^28^ During molecular dynamics simulations, the relative positions of compounds and mTyr are in constant flux, resulting in dynamic changes in the number of hydrogen bonds formed between them. As seen in Fig. 4F and S7F,† the number of hydrogen bonds for compounds 3a–3e fluctuates between 0 and 3, with occasional bond ruptures. Specifically, compounds 3a, 3b, and 3c maintain 1 hydrogen bond with mTyr for most of the simulation time, while compounds 3d and 3e stabilize at 1–2 hydrogen bonds. This trend closely aligns with the results from molecular docking.

In conclusion, molecular dynamics simulations provide a comprehensive understanding of the dynamic behaviour and stability of compound–mTyr complex systems. Evaluation parameters such as RMSD, RMSF, *R*_g_, SASA, and hydrogen bond analysis shed light on the interactions between small molecule compounds and the mTyr enzyme. These insights contribute to the design and synthesis of highly effective mTyr inhibitors.

### Cytotoxicity assay of compound 3b

#### Cytotoxicity analysis on human epidermal melanoma A375 cells

**Fig. 5:**
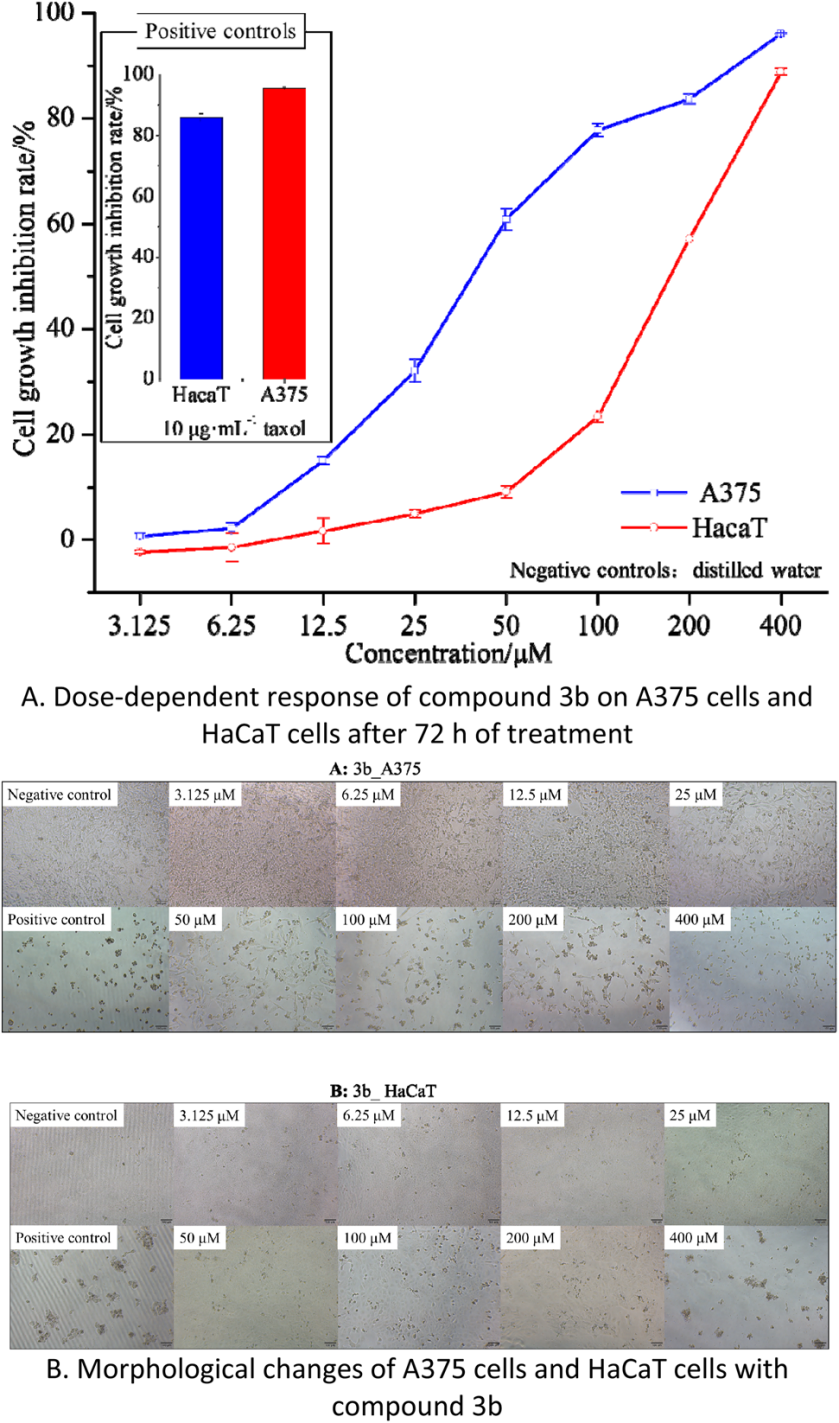
The effects of compound 3b on cell proliferation and cytotoxicity in A375 and HaCaT cells.

The abnormal metabolism of mTyr in the human body is closely associated with the development and treatment of melanoma, a form of skin cancer. Therefore, the discovery of compounds with strong mTyr inhibitory activity from those possessing such activity is highly promising for combating melanoma.^29^ Cell proliferation and cytotoxicity assays serve as fundamental data for evaluating the efficacy of compounds and determining safe concentrations for further applications. Based on a thorough analysis of the activity and inhibition mechanisms of compounds 3a–3e, compound 3b, which exhibited the highest mTyr inhibitory activity, was selected. Using human epidermal melanoma A375 cells as an *in vitro* cellular model, the preliminary assessment of its cytotoxicity was conducted by monitoring cell numbers and observing cell growth patterns, as depicted in the Fig. 5A.

The results indicate that compound 3b has IC_50_ values of 40.77 μM and 168.60 μM against A375 and HaCaT cells, respectively. This suggests that compound 3b exhibits significant toxicity towards A375 cells while displaying minimal toxicity towards HaCaT cells. As illustrated in the Fig. 5B, within the concentration range of 3.125–25 μM, compound 3b exerts negligible toxicity on HaCaT cells, with an inhibition rate below 5%, and there are no significant morphological changes observed. In contrast, compound 3b demonstrates substantial toxicity against A375 cells, with an inhibition rate of 32.12%. This is characterized by reduced cell–cell contacts, chromatin condensation, and a crescent-shaped nuclear membrane. When the concentration is increased to 50 μM, compound 3b exhibits an inhibition rate of 9.09% on HaCaT cells, resulting in only minor alterations in cell morphology. However, at the same concentration, it exerts significantly greater toxicity on A375 cells, with an inhibition rate of 60.89%. This leads to pronounced changes in cell morphology, including loss of cell–cell contacts, membrane vesicle formation, and the presence of individual apoptotic bodies.^30^

These findings indicate that within the concentration range of 0–25 μM, compound 3b does not exhibit significant toxicity towards HaCaT cells but displays substantial toxicity towards A375 cells. Consequently, compound 3b shows potential as an anti-melanoma agent. However, further research is required to explore strategies for maintaining its toxicity against A375 cells while reducing its impact on HaCaT cells.

## Experimental section

### Materials, cell lines, and insects

Commercially available 4-hydroxycinnamic acid, thionyl chloride (SOCl_2_), hydroquinone, cinnamoyl chloride, phenylpropionyl chloride, benzoyl chloride, 4-benzoyl chloride, l-tyrosine, l-3,4-dihydroxyphenylalanine (l-DOPA), and kojic acid were products of Adamas-Beta purchased from Shanghai Titan Scientific Co., Ltd (Shanghai, China) and used directly without further purification. 4-Hydroxycinnamoyl chloride was synthesized according to ref. 13. Tyr from mushroom were products of Macklin purchased from Shanghai Macklin Biochemical Co., Ltd (25KU, Shanghai, China). Taxol was obtained from Molecular Probes (Eugene, USA). Thin-layer chromatography (TLC) silica plates (GF254) and silica gel (300–400 mesh) were purchased from Qingdao Marine Chemical, Co., Ltd (Qingdao, China), and the spots were detected under UV light. Other reagents and organic solvents were purchased from Shanghai Titan Scientific Co., Ltd (Shanghai, China), unless otherwise stated. All reagents used were of analytical grade or higher and were used directly without further purification.

A375 Human melanoma (A375) tumor cells and human immortalized keratinocyte (HaCaT) non-tumor cells were obtained from the Nanjing Pusheng Biomedical Technology Co., Ltd (Jiangsu, China). Dulbecco's modified Eagle's medium (DMEM) and fetal bovine serum (FBS) were obtained from GIBCO (New York, USA). Both A375 tumor cells and HaCaT non-tumor cells were cultured in DMEM supplemented with 10% (v/v) FBS (New York, USA) at 37 °C with 5% CO_2_ in a humidified incubator. Cell counting kit-8 (CCK-8) solution was obtained from Dojindo Laboratories (Tokyo, Japan).

### Synthesis

A quantity of 1.00 g (9.10 mmol) of hydroquinone was introduced into a 100 mL round-bottom flask containing 25.0 mL of dichloromethane (CH_2_Cl_2_, DCM) under ice-bath conditions. After stirring for 5 minutes, 1.10 g (10.92 mmol) of triethylamine (TEA) was added to the mixture, followed by the slow addition of substituted cinnamoyl chloride 2a–2e (10.01 mmol) dissolved in 15 mL of mL DCM. The reaction mixture was shielded with argon gas and monitored by TLC using ethyl acetate/petroleum ether as the mobile phase. Once the reaction was completed, the mixture was quenched with 25.0 mL of saturated sodium bicarbonate (NaHCO_3_), and then extracted with ethyl acetate (20.0 mL × 2), followed by washing with 20.0 mL of distilled water and 20.0 mL of saturated saline solution. After drying with anhydrous sodium sulfate, the organic extracts were evaporated under reduced pressure to remove the solvent, and the resulting mixture was further purified by silica gel column chromatography eluting with which yielded compounds 3a–3g.

### Structural characterization

^1^H NMR (500 MHz) and ^13^C NMR (126 MHz) were measured on a Bruker Avance II 500 MHz spectrometer using chloroform (CDCl_3_) and methanol (CD_3_OD) as solvents. HREIMS was carried out using Thermo Scientific Q Exactive (Waltham, USA). UV-visible spectra were obtained using UV-2600 spectrophotometer (Shimadzu, Japan) scanning from 240 to 450 nm. Melting points were determined using a X-4 digital display melting point apparatus (Shanghai, China) and are uncorrected.

### Inhibitory activity assay

The synthesized hydroquinone ester derivatives were dissolved in 3% dimethyl sulfoxide (DMSO) solution and then diluted with phosphate-buffered solution (PBS, pH 6.86, 100 mM) to obtain different concentrations. In a 96-well plate, 40 μL of the test compound solution, 100 μL of PBS, and 40 μL of mTyr solution were added and mixed sequentially. The plate was incubated at 37 °C for 30 minutes, after which 20 μL of l-tyrosine solution (1 mM) was added and mixed. The absorbance at a wavelength of 475 nm was measured using a SpectraMax 190 microplate reader (MolecularDevices, USA) continuously for 30 minutes. PBS was used as a blank control, and kojic acid was used as a positive control. The mTyr activity inhibition rate was calculated using eqn (1), and the inhibitory effect of cinnamic acid ester derivatives on mTyr was expressed as the concentration that inhibited 50% of the mTyr activity inhibition rate (IC_50_).^31^

1 mTyr activity inhibition rate (%) = [(*A* − *B*) − (*C* − *D*)]/(*A* − *B*) × 100%

where *A* is the absorbance of the blank control after incubation, *B* is the absorbance of the blank control before incubation, *C* is the absorbance of the test compound solution after incubation, and *D* is the absorbance of the test compound solution before incubation.

### Inhibition reversibility and type assay

The methodology for evaluating the reversibility of inhibition and the inhibition type of the compounds on mTyr closely parallels the procedure outlined in the inhibitory activity assay of compounds on mTyr.

For the evaluation of inhibition reversibility, a constant concentration of 1 mM l-tyrosine was employed. Various mTyr concentrations (100, 200, and 300 U mL^−1^) were applied, accompanied by compounds 3a–3e concentrations that will be determined based on the IC_50_ (OD_475_ × min^−1^) value. The absorption was measured to determine the initial enzymatic reaction rate corresponding to each enzyme concentration. Specifically, the initial enzymatic reaction rate was calculated by subtracting the initial absorbance from the absorbance recorded at a specific time and then dividing the result by the corresponding time interval.^32^ Subsequently, the collected data were plotted on a scatter plot, with the initial enzymatic reaction rate represented on the *Y*-axis and the enzyme concentration on the *X*-axis. In cases of reversibility, this will result in fitting a set of straight lines passing through the origin. Conversely, when reversibility was absent, a set of parallel lines emerged as the fit.^33^

In order to investigate the inhibition type, a final concentration of 300 U per mL mTyr was employed, accompanied by varying concentrations of l-tyrosine. The enzymatic reaction rate was evaluated for different concentrations of compounds 3a–3e. Subsequently, the ΔOD_475_ values were plotted against the l-tyrosine concentration using the Lineweaver–Burk equation, allowing for the creation of double reciprocal plots. The intersection points of these lines revealed the nature of mTyr inhibition caused by the compounds.^34^

### UV-visible spectroscopy analysis

The chelation between hydroquinone ester derivatives and the copper(ii) ion active center of mTyr was investigated using a UV-2600 spectrophotometer (Shimadzu, Japan), with some modifications based on a previously published method.^32^ For the assay, 1.0 mg of the sample was dissolved in 10 mL of PBS. The concentration of copper sulfate (CuSO_4_) in the PBS was set at 0.125 mM, and the mTyr concentration was adjusted to 300 U mL^−1^. All working solutions were made up to a final volume of 3 mL using PBS, ensuring consistency across all samples. After gentle mixing with a vortex shaker, the samples were incubated at 30 ± 2 °C for 10 minutes with intermittent shaking. Subsequently, the UV-visible spectrophotometer was employed to measure the absorption spectrum in the range of 240 nm to 500 nm.^35^

### Molecular docking and molecular dynamics studies

For molecular docking, the three-dimensional structure of the mTyr protein 2Y9X was firstly obtained from the Protein Data Bank (PDB). Then the protein structure was processed using UCSF Chimera 1.16, where H_2_O molecules were removed, redundant chains were deleted, and the AMBER ff14SB force field was applied. The small molecule ligands were drawn using ChemOffice 2018 and converted into three-dimensional conformations using Chem3D, followed by energy minimization and saving the structures in the mol2 format. To construct the active site, the crystallographic ligand positions from the protein structure were used as references. The active site was defined with the centre coordinates at (*x* = −10.02, *y* = −28.82, *z* = −43.59), and a box of dimensions 25 × 25 × 25 Å was created around it. The AutoDock Vina software was utilized to perform docking calculations, with the default settings.^36^

After the molecular docking, pre-equilibration involved NVT and NPT simulations, followed by a 50 000 ps molecular dynamics simulation using GROMACS. LINCS algorithm constrained hydrogen bonds, SETTLE maintained water molecules, and Parrinello–Rahman controlled pressure. The PME method was used for long-range electrostatic interactions. Trajectory files provided crucial data on complex properties.^37^

### Cell cytotoxicity study

Cell proliferation and cytotoxicity assays were performed using the CCK-8 on Human Epidermal Melanoma A375 and HaCaT cells. Initially, the cells were seeded in 96-well plates at a density of 4 × 10^4^ cells per well and incubated for 24 hours at 37 °C with 5% CO_2_ in a humidified incubator. Each treatment was conducted in six duplicate wells. Various concentrations of the tested compounds were added to the wells, and the cells were further incubated for 72 hours. After the incubation period, CCK-8 solution was added to each well and incubated for an additional 2 hours. The absorbance of each well at 450 nm was measured using an absorbance microplate reader (BioTek ELx800, USA). Negative controls with cells treated with distilled water and positive controls with cells were treated with 10 μg mL^−1^ taxol was included in the assay. The percentage of cell growth inhibition was calculated using eqn (2).2
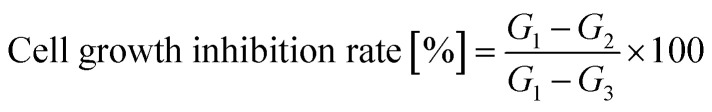
where *G*_1_ represents the absorbance of a mixture containing cells, medium, and CCK8, *G*_2_ represents the absorbance of absorbance of a mixture containing cells, medium, CCK8, and the test compound; *G*_3_ represents a mixture containing medium and CCK8.

Cell growth and morphology were observed using an inverted microscope (Olympus IX51, Japan). The experiments were conducted in triplicate to ensure accuracy and consistency of results.

### Statistical analysis

All data obtained from cultured cells were analysed by one-way analysis of variance (ANOVA) followed by post hoc Bonferroni's multiple comparison test. In some cases, Student's *t*-test was applied. Analyses were carried out using the Prism 4.0 statistics program (GraphPad, San Diego, CA). Differences were considered significant at *p* < 0.05.

## Conclusions

Tyrosinase are a diverse class of proteins with a wide range of physiological functions and are crucial enzymes in melanin biosynthesis. Focusing on the excellent inhibitory activity of hydroquinone against tyrosinase and the controversial safety concerns, this study employed a scaffold hybridization strategy to synthesize hydroquinone–benzoyl ester derivatives, aiming to develop novel, highly efficient, and low-toxicity tyrosinase inhibitors. In this research, we synthesized a series of hydroquinone–benzoyl ester analogs (3a–3g). *In vitro* inhibition results indicated that these compounds all exhibited inhibitory activity against mTyr, with compounds 3a–3e displaying particularly good inhibition. We further elucidated the molecular mechanisms of compounds 3a–3e in inhibiting mTyr through inhibition kinetics, UV spectroscopy analysis, and molecular simulations. The results revealed that the inhibition process of compounds 3a–3e on mTyr was reversible, and they did not chelate with the copper ions in the active center of enzyme. However, the inhibition mechanisms varied, closely related to the presence or absence of double bonds and phenolic hydroxyl groups in the chemical structures. Molecular docking simulations and molecular dynamics analysis further supported these experimental findings. To explore the potential applications of these compounds, we selected the most active compound 3b, and conducted toxicity tests on melanoma cells. In cell toxicity experiments, compound 3b exhibited high toxicity to A375 cells, while displaying low toxicity to HaCaT cells, with a dose-dependent effect. This study provides promising candidate compounds for the development of tyrosinase inhibitors with broad application prospects.

## Ethical statement

This study does not involve the use of human or animal subjects, and therefore, ethical approval and informed consent are not applicable to this study.

## Author contributions

Conceptualization, X. Q. Yang, Y. P. He, and P. Zhao; methodology, X. Q. Yang and D. Xie; software, K. J. Han; validation, D. Xie and K. J. Han; formal analysis, Q. Jiang; investigation, D. Xie and K. J. Han; resources, J. L. Zhou; data curation, Dong Xie and K. J. Han; writing—original draft preparation, D. Xie; writing—review and editing, S. D. Xie, Y. J. Zhang and P. Zhao; visualization, K. J. Han; supervision, X. Q. Yang; project administration, X. Q. Yang; funding acquisition, D. Xie; Q. Jiang; J. M. Xu; P. Zhao; X. Q. Yang. All authors have read and agreed to the published version of the manuscript.

## Conflicts of interest

The authors declare no conflict of interest.

## Supplementary Material

RA-014-D4RA00007B-s001
