# Supplementary material for: Design, synthesis, and inhibitory activity of hydroquinone ester derivatives against mushroom tyrosinase
Source: RSC Adv. 2024 Feb 16;14(9):6085–95. doi: 10.1039/d4ra00007b (PMC10870825; doi:10.1039/d4ra00007b)
Supplement: RA-014-D4RA00007B-s001 [file RA-014-D4RA00007B-s001.pdf]

Electronic Supplementary Material (ESI) for RSC Advances.
This journal is © The Royal Society of Chemistry 2024

Supporting Information

Design, Synthesis, and Inhibitory Activity of Hydroquinone Ester Derivatives against Mushroom Tyrosinase

Dong Xie[a, #], Kangjia Han[a, #], Qian Jiang[a], Sida Xie[a], Jielong Zhou[a], Yingjun Zhang[b], Junming Xu[c], Yuanping He[d], Ping Zhao[e,*] , and Xiaoqin Yang[a,*]

[a]Key Laboratory of National Forestry and Grassland Administration on Highly-Efficient Utilization of Forestry Biomass Resources in Southwest China, Southwest Forestry University, Kunming Yunnan 650224, PR China

[b]Laboratory of Phytochemistry and Plant Resources in West China, Kunming Institute of Botany, Chinese Academy of Sciences, Kunming Yunnan 650204, PR China

[c]Institute of Chemical Industry of Forest Products, Chinese Academy of Forestry, Nanjing Jiangsu 210042, PR China

[d]Kunming Beiye Dai Medicine Research Institute, Kunming Yunnan 650499, PR China

[e]Key Laboratory of Ministry of Education for Forest Resources Conservation and Utilization in the Southwest Mountains of China, Southwest Forestry University, Kunming Yunnan 650224, PR China

[#]Co-first authors. These authors contributed equally to this work.

*Corresponding Authors.

hypzhao2022@163.com (P. Zhao)

yangxiaoqin@swfu.edu.cn (X.Q. Yang)

This file includes 7 Figures and 2 Tables

Contents of the Figures and the Tables

Figure S1. ^{1}H NMR and ^{13}C NMR for compound 3a-3g

Figure S2. HRMS for compound 3a-3g

Figure S3. The inhibitory activity of compound 3a-3g on mTyr

Figure S4. Inhibition reversibility (A) and inhibition type (B) of compound 3a, 3c-3e on mTyr.

Figure S5. UV Spectra of compound 3a, 3c-3e before and after interaction with mTyr

Figure S6. The crystal structure of mTyr (PDB ID: 2Y9X)

Figure S7. Docking model for compound 3a, 3c-3e with mTyr (A) and Molecular dynamics results of compound 3a, 3c-3e and (3a, 3c-3e) -mTyr complex with: B. RMSD, C. RMSF, D. Rg, E. SASA, and F. H-bonds

Table S1. Linear fitting equation, Michaelis constant (K_m), maximum reaction rate (V_m), and inhibition type for mTyr at varying concentrations of compounds 3a-3e

Table S2. Docking energy and bonding condition of compounds 3a-3e with mTyr

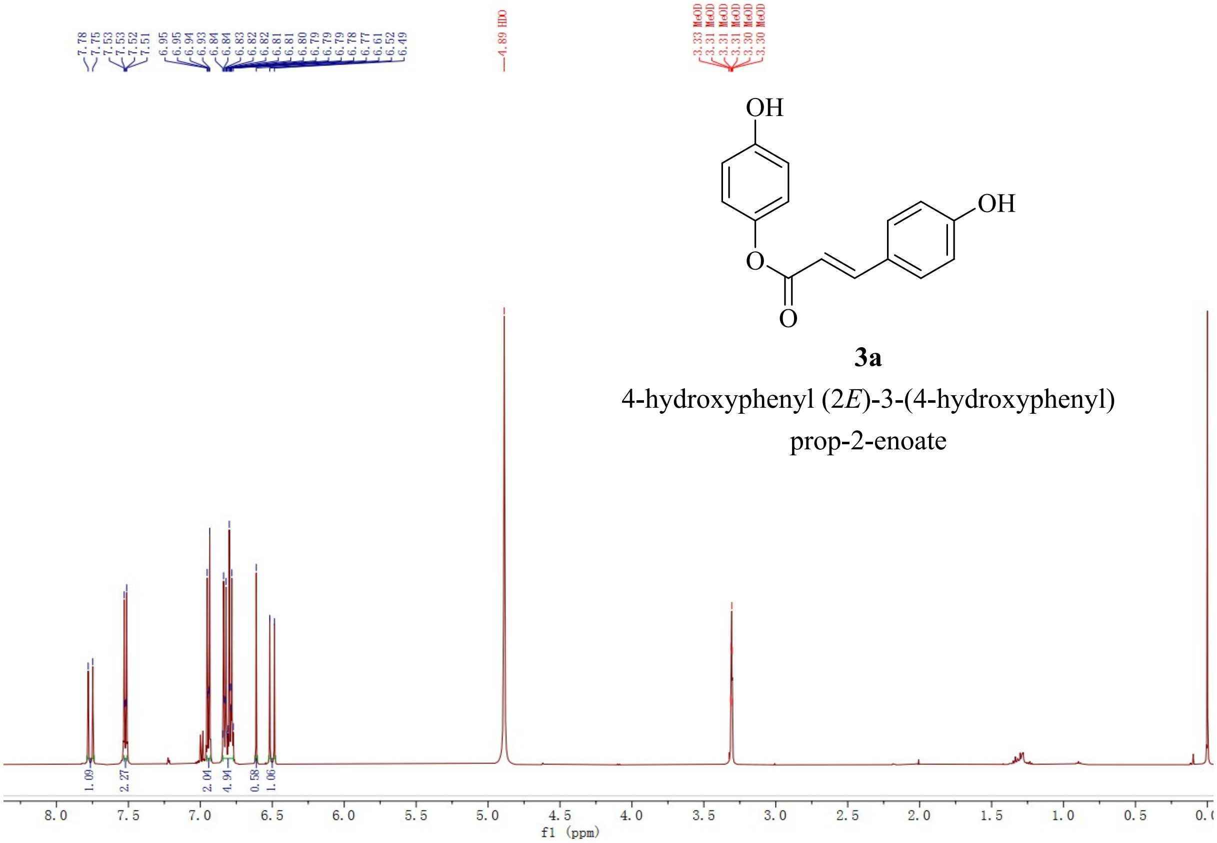

(1) ^{1}H NMR for compound 3a

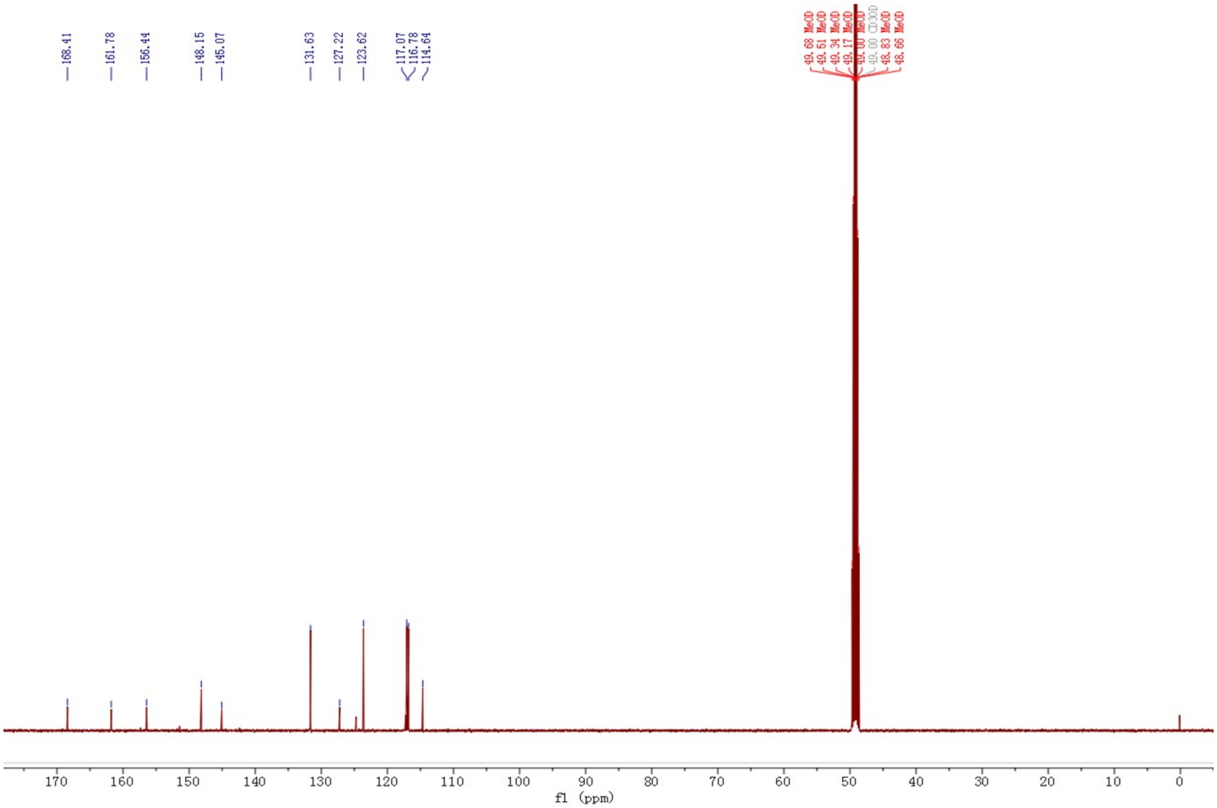

(2) ^{13}C NMR for compound 3a

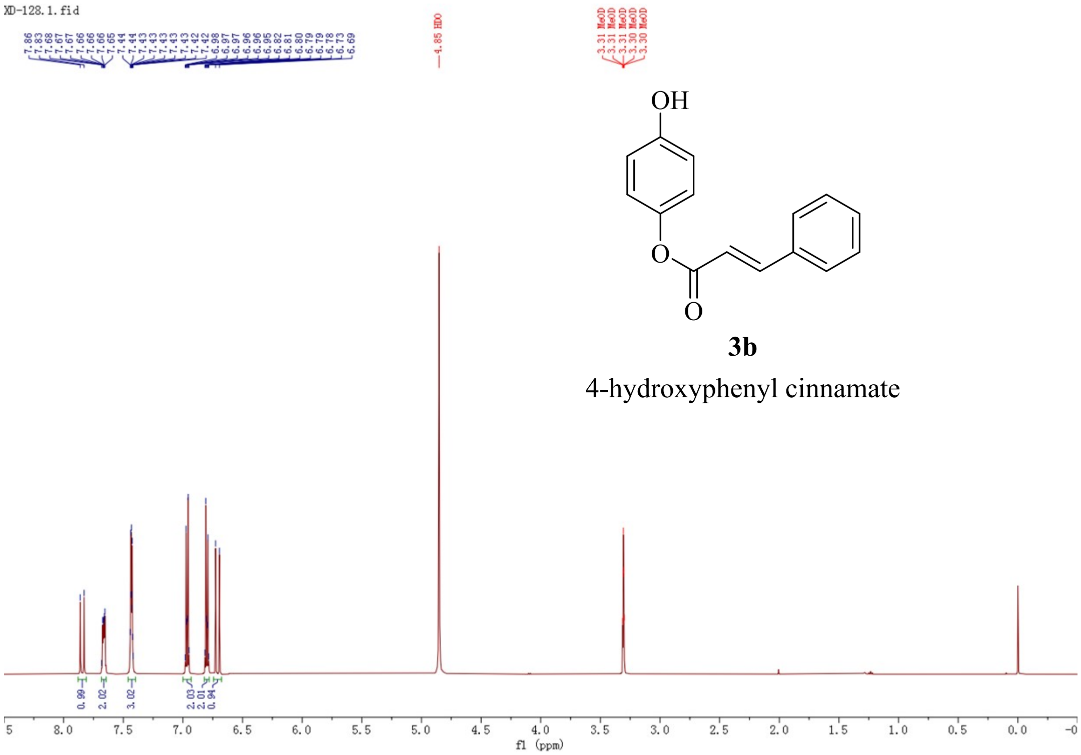

(3) ^{1}H NMR for compound 3b

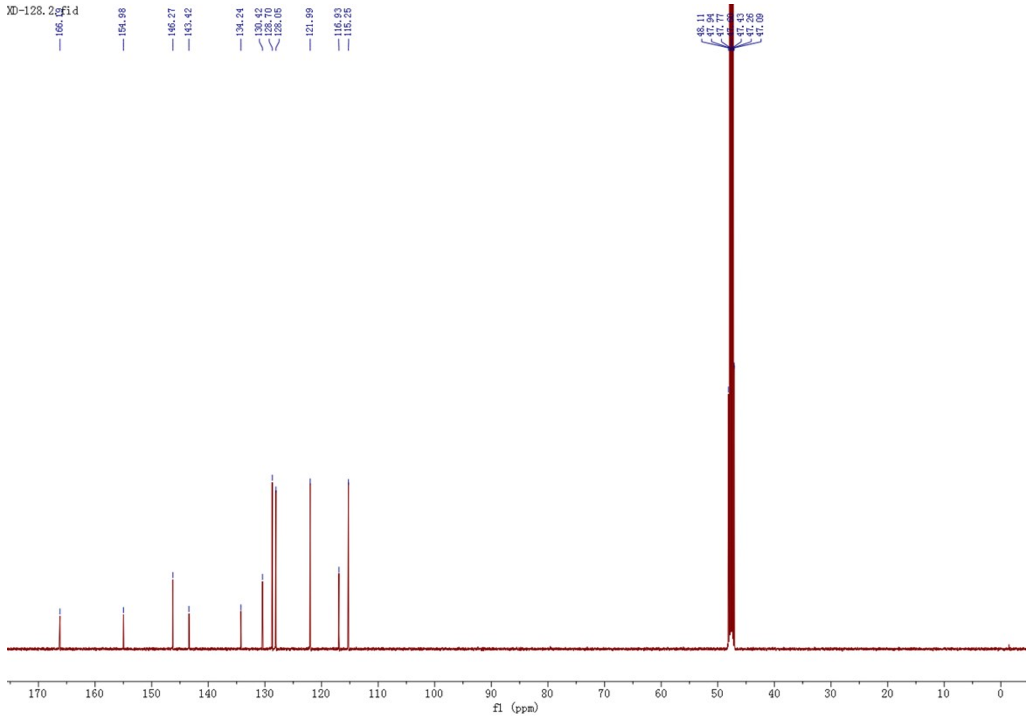

(4) ^{13}C NMR for compound 3b

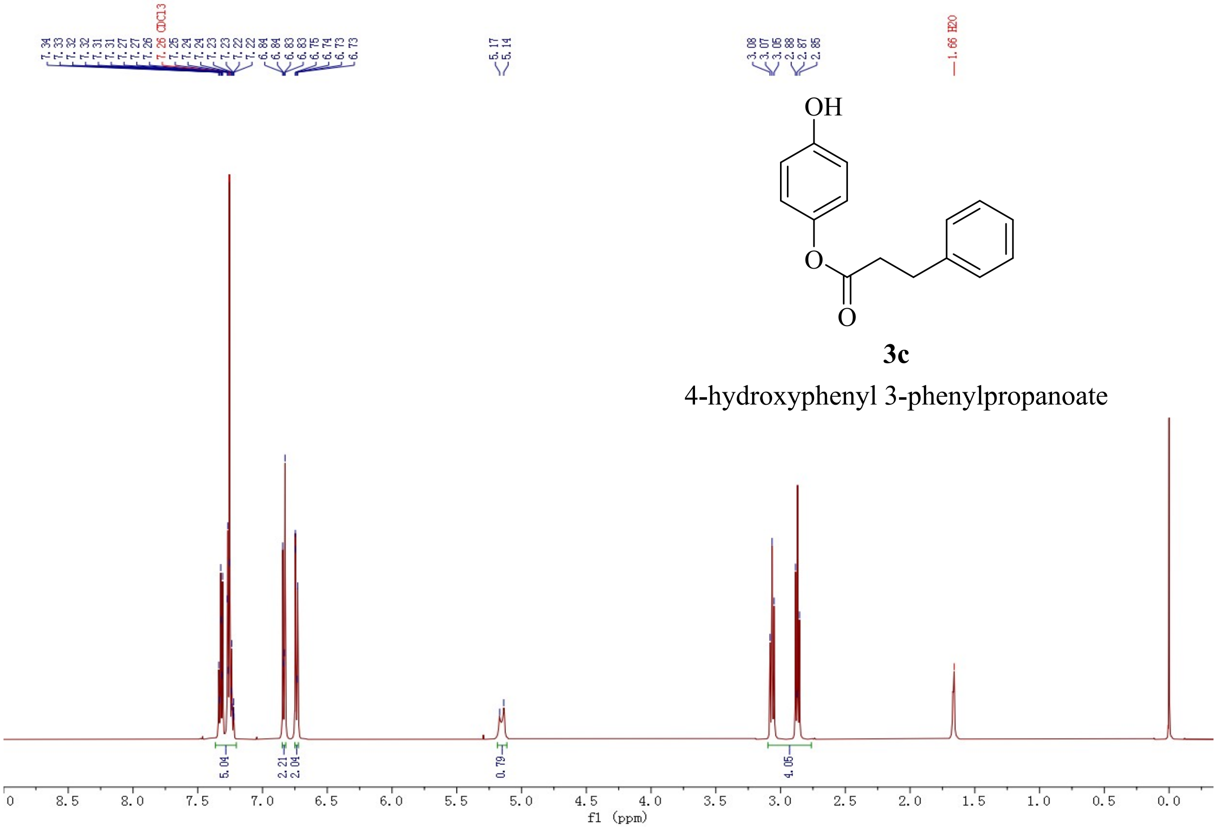

(5) ¹H NMR for compound 3c

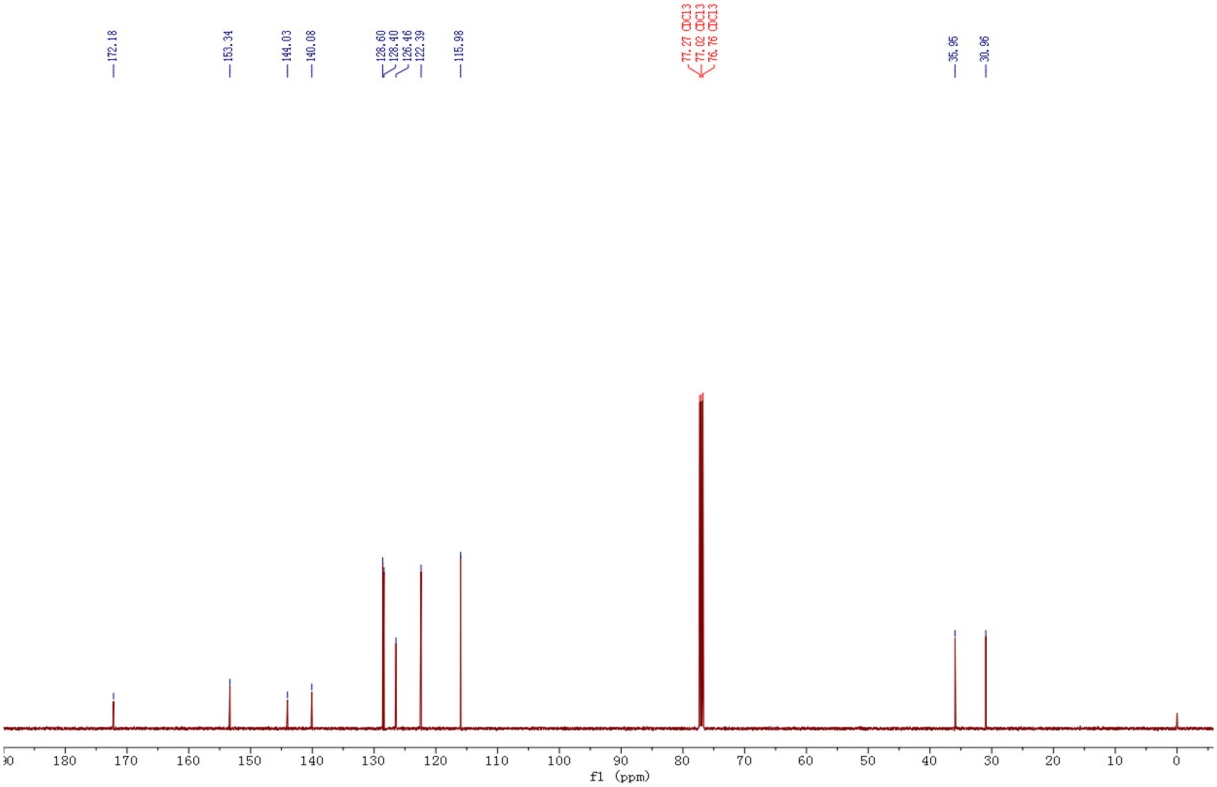

(6) ¹³C NMR for compound 3c

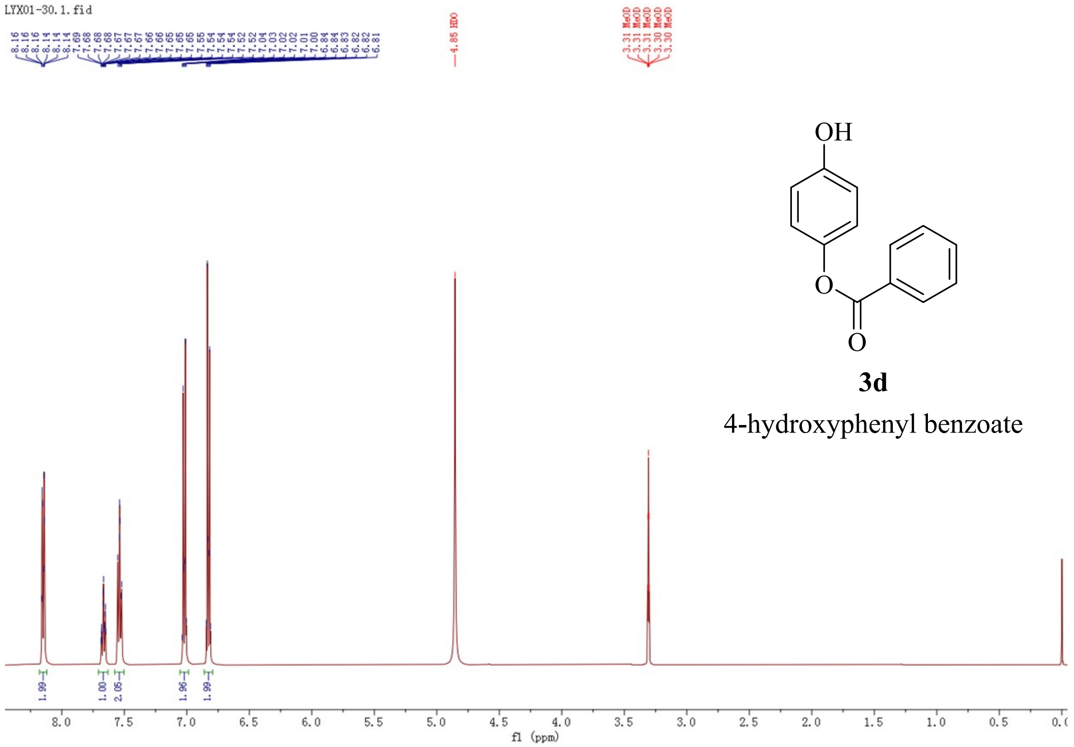

(7) ^{1}H NMR for compound 3d

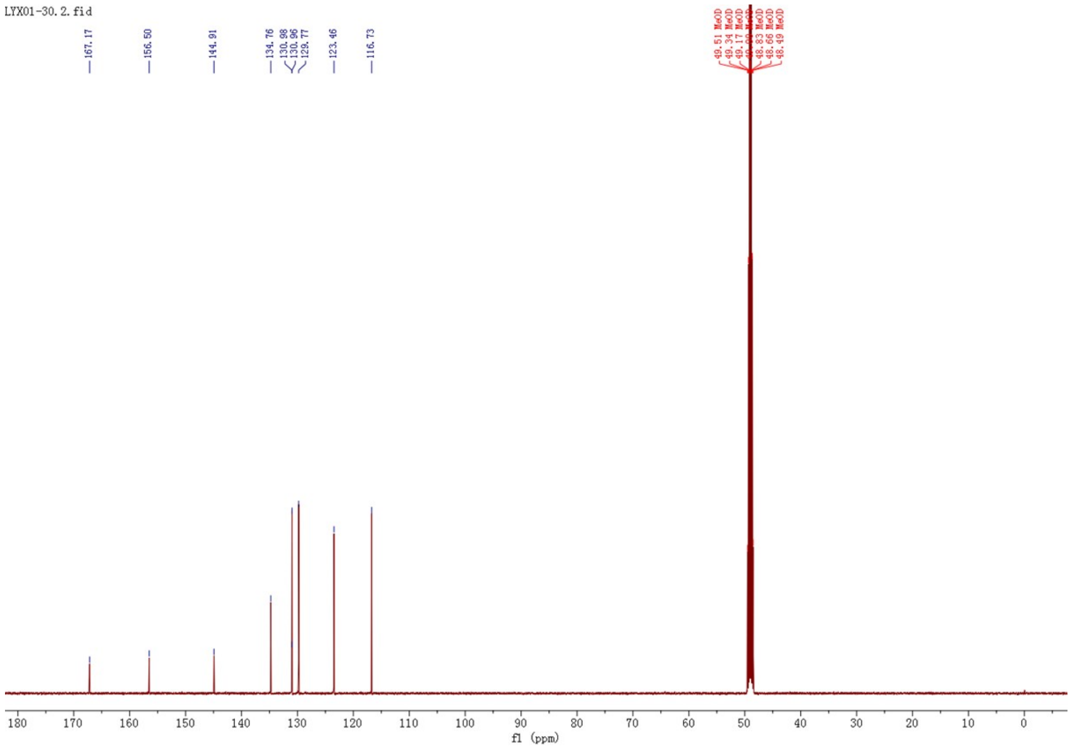

(8) ^{13}C NMR for compound 3d

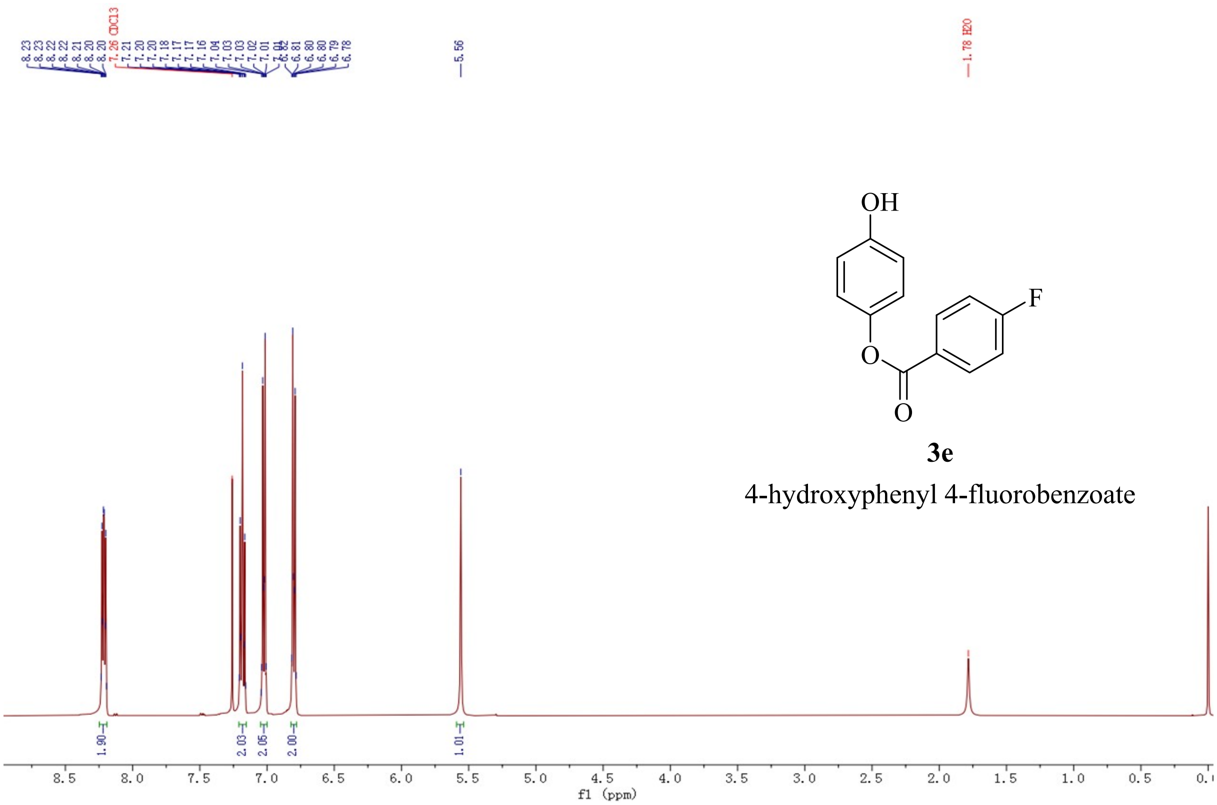

3e

4-hydroxyphenyl 4-fluorobenzoate

(9) ^{1}H NMR for compound 3e

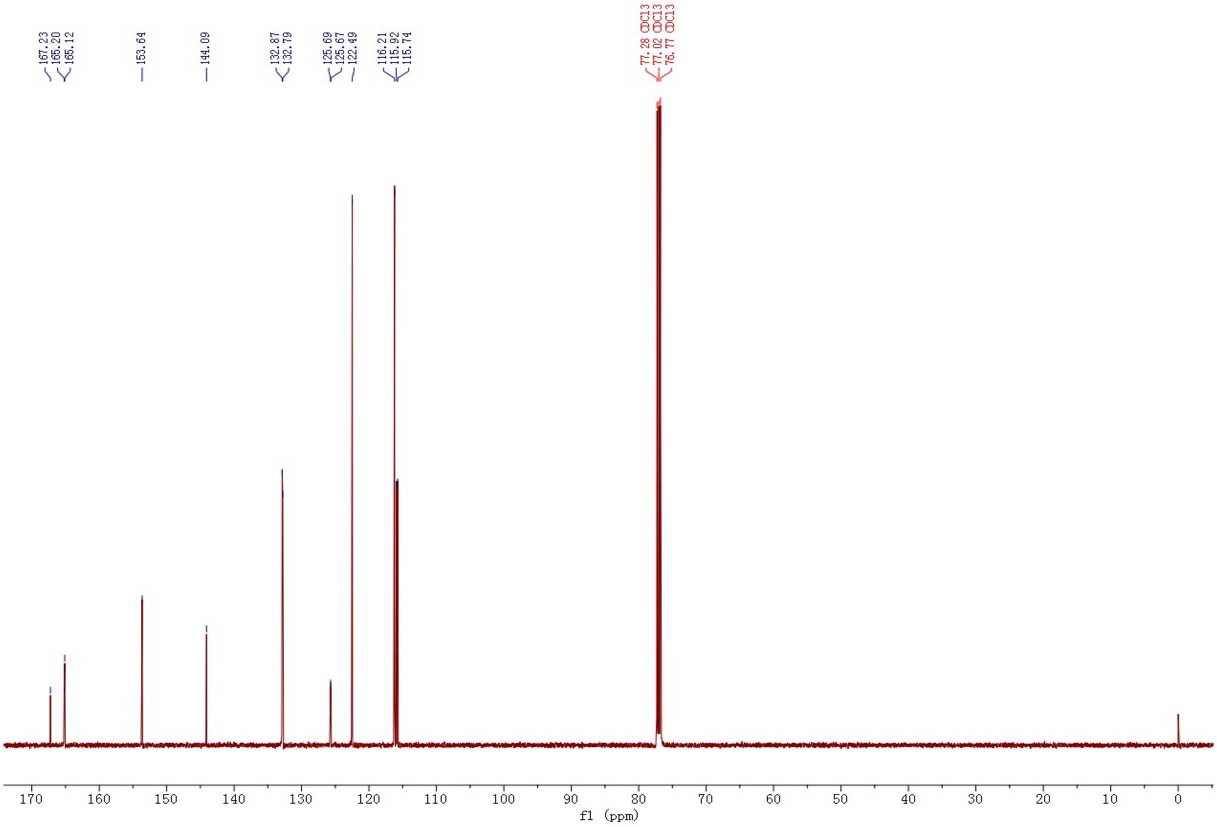

(10) ^{13}C NMR for compound 3e

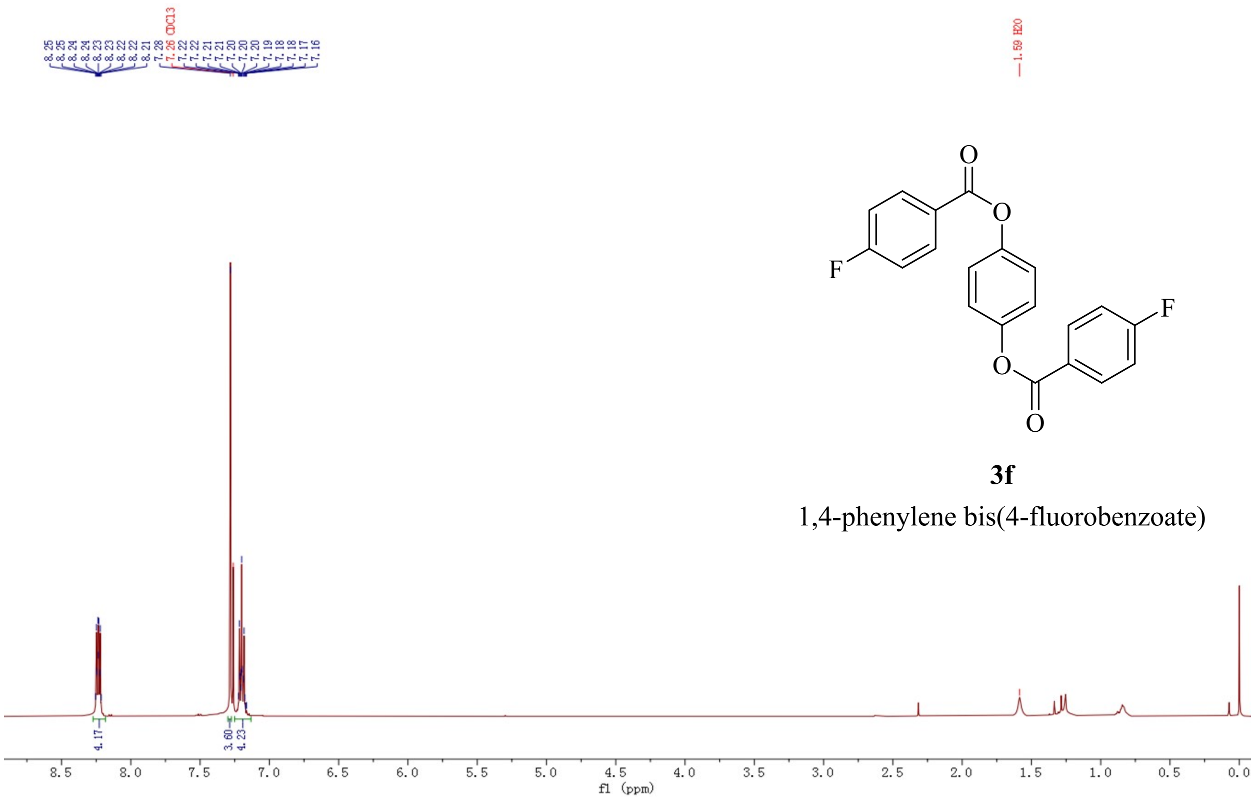

(11) ^{1}H NMR for compound 3f

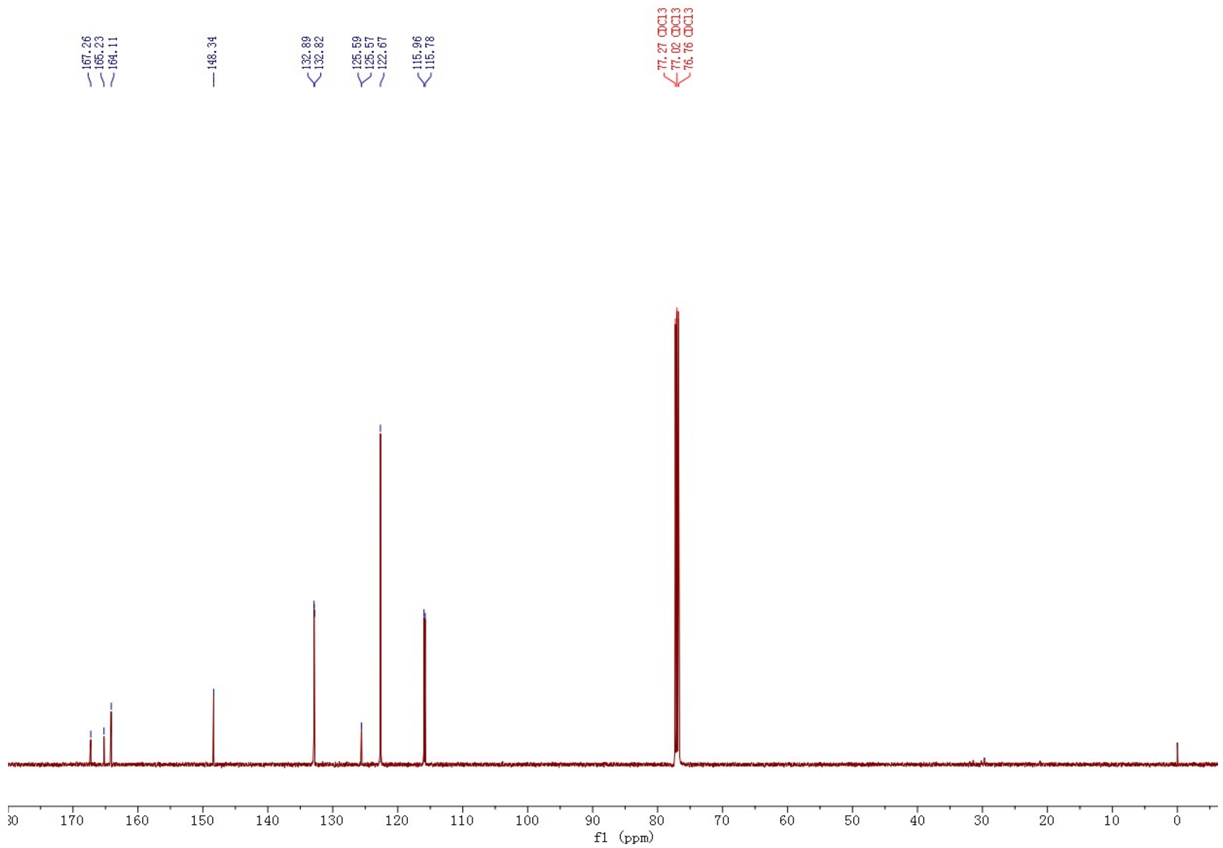

(12) ^{13}C NMR for compound 3f

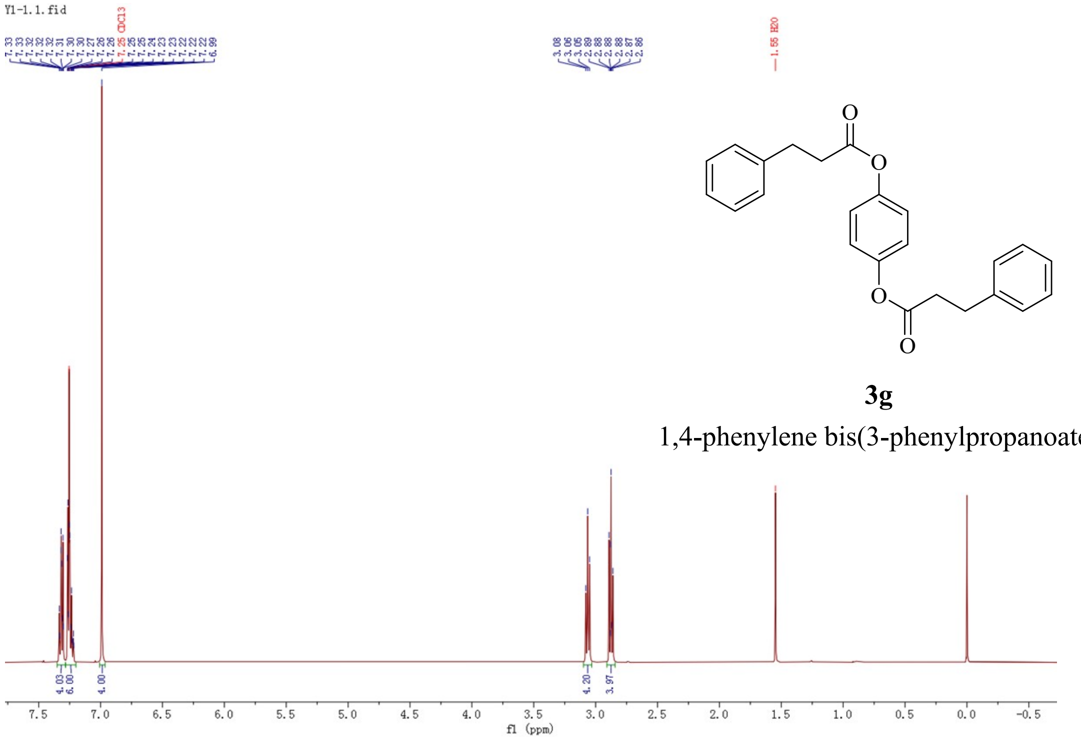

3g

1,4-phenylene bis(3-phenylpropanoate)

(13) ^{1}H NMR for compound 3g

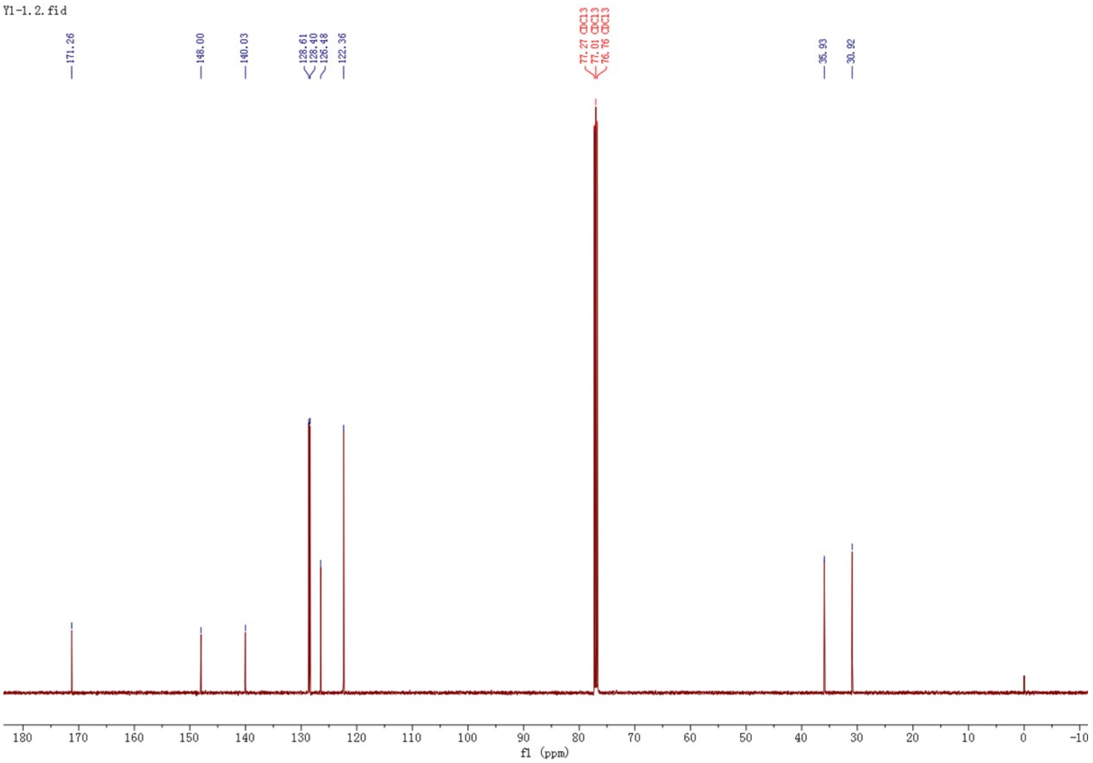

(14) ^{13}C NMR for compound 3g

Figure S1. ^{1}H NMR and ^{13}C NMR for compound 3a-3g

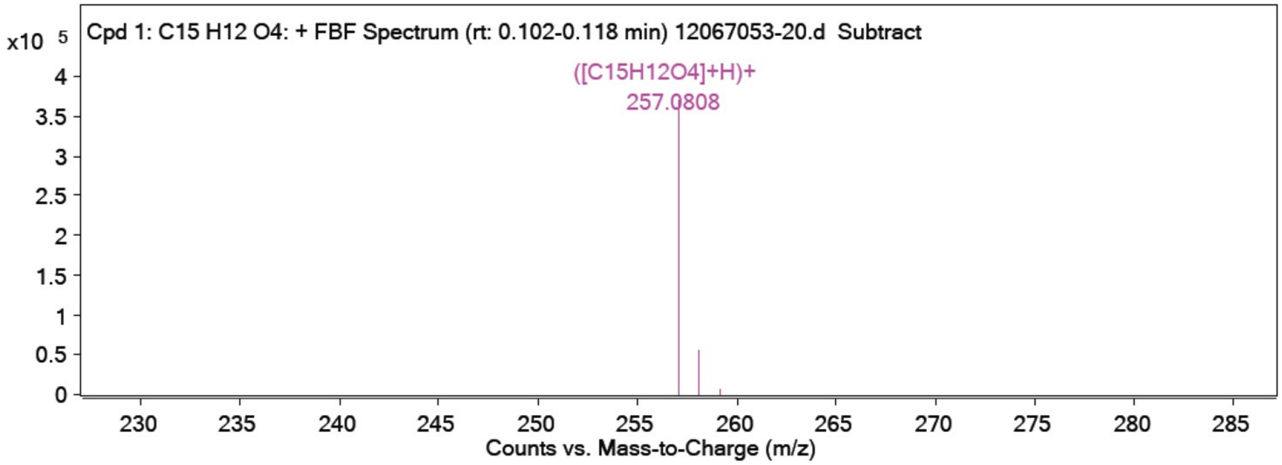

(1) HRMS for compound 3a

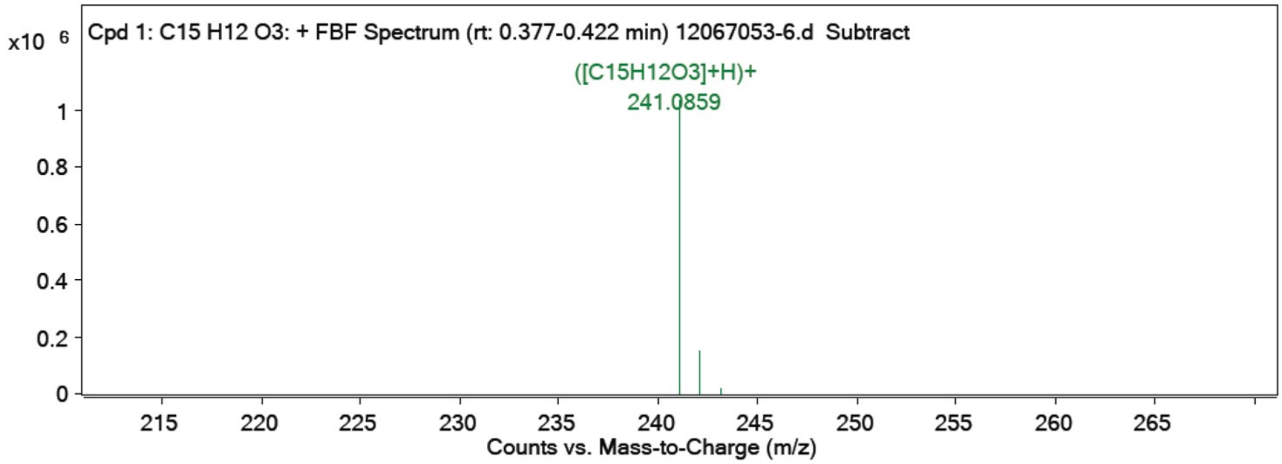

(2) HRMS for compound 3b

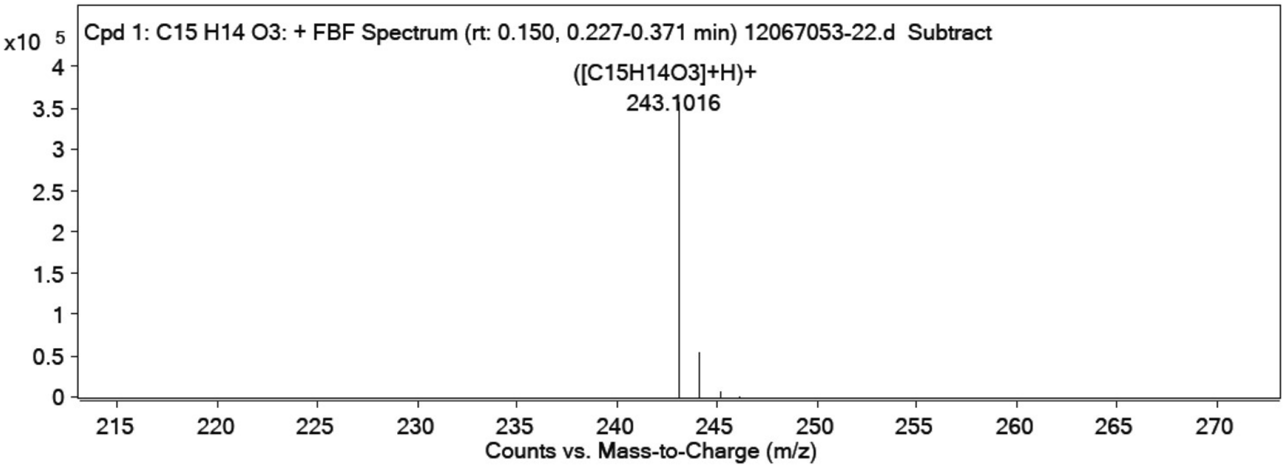

(3) HRMS for compound 3c

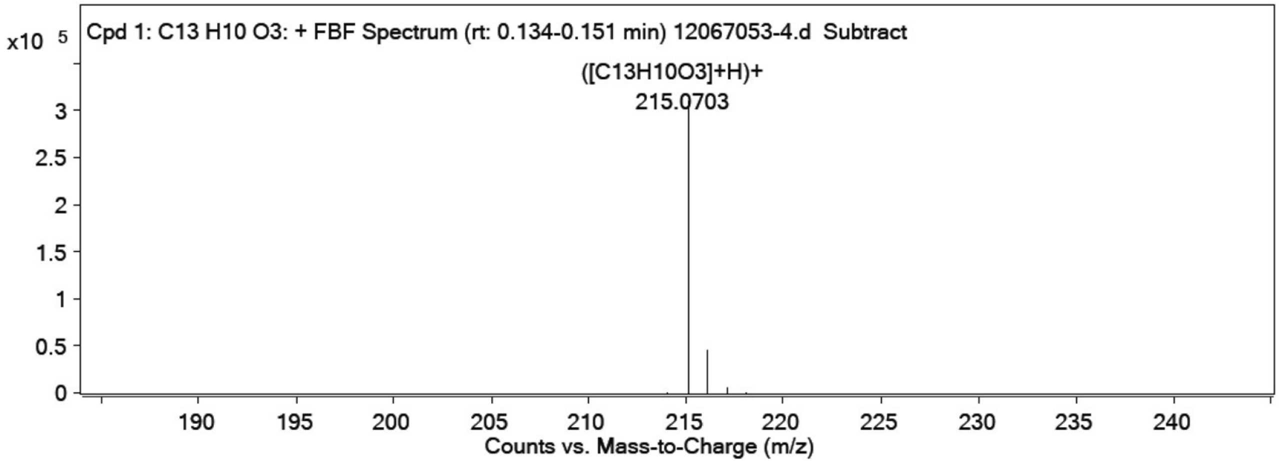

(4) HRMS for compound 3d

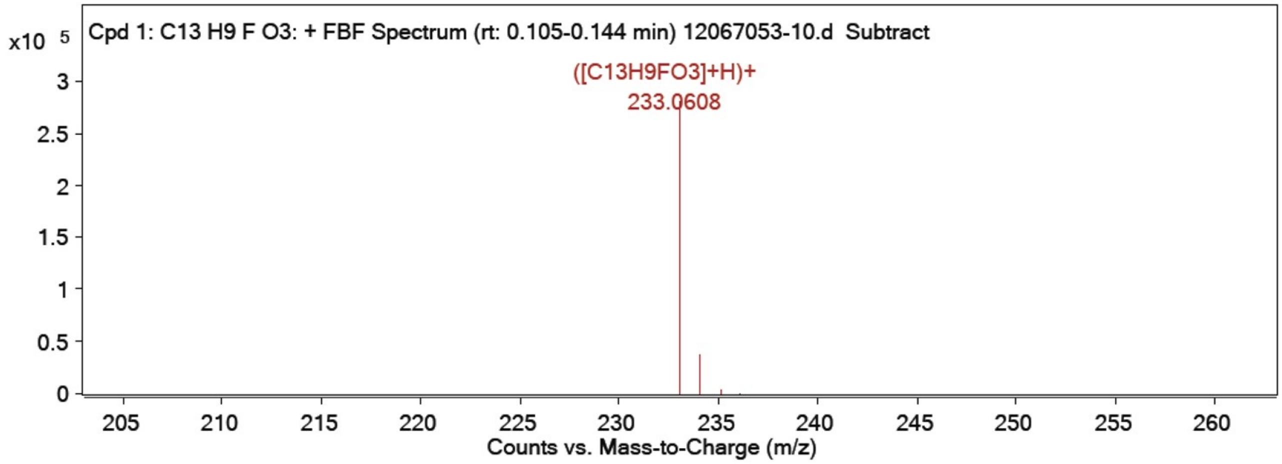

(5) HRMS for compound 3e

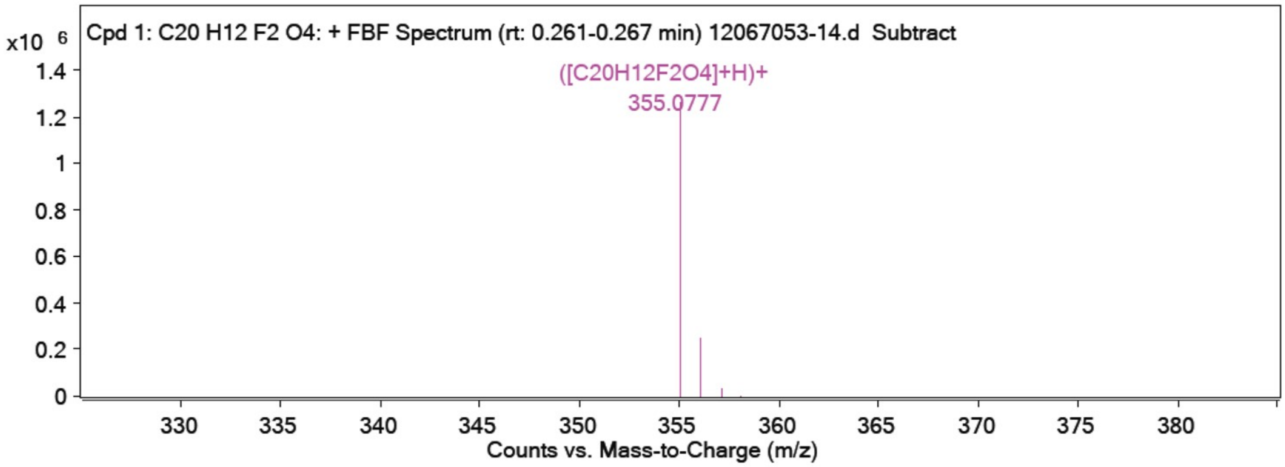

(6) HRMS for compound 3f

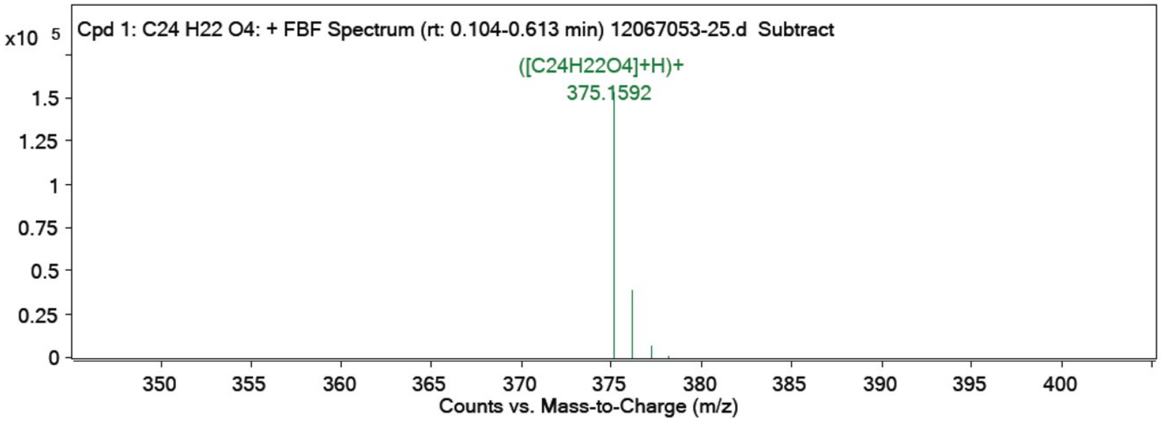

(7) HRMS for compound 3g

Figure S2. HRMS for compounds 3a-3g

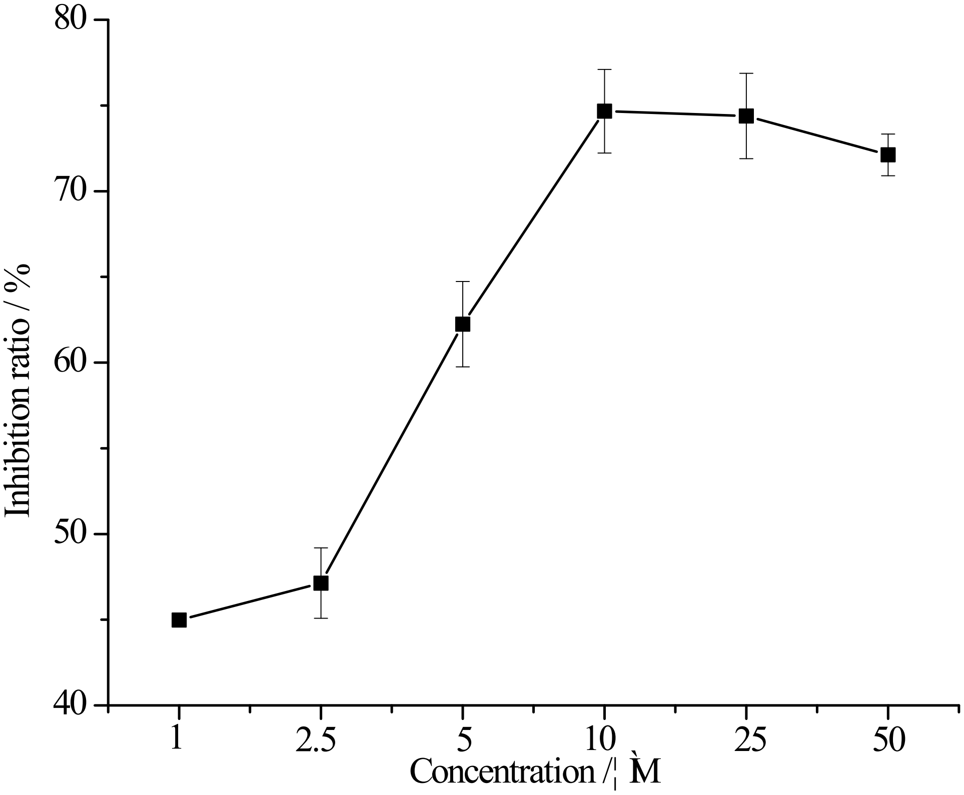

(1) The inhibitory activity of compound 3a on mTyr

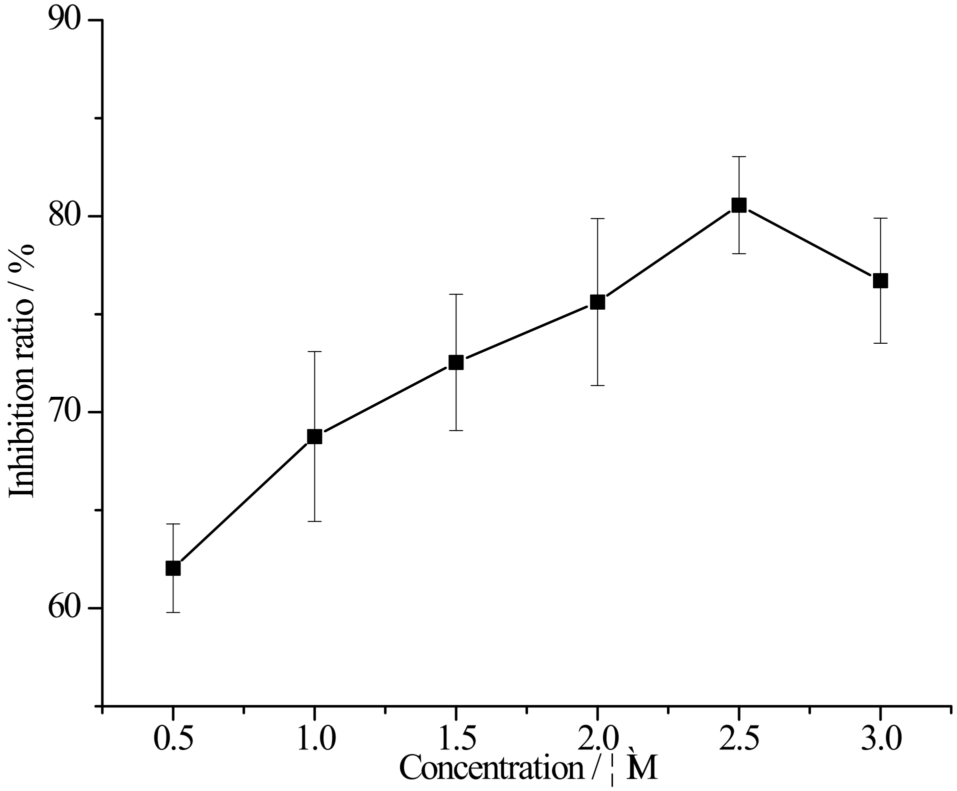

(2) The inhibitory activity of compound 3b on mTyr

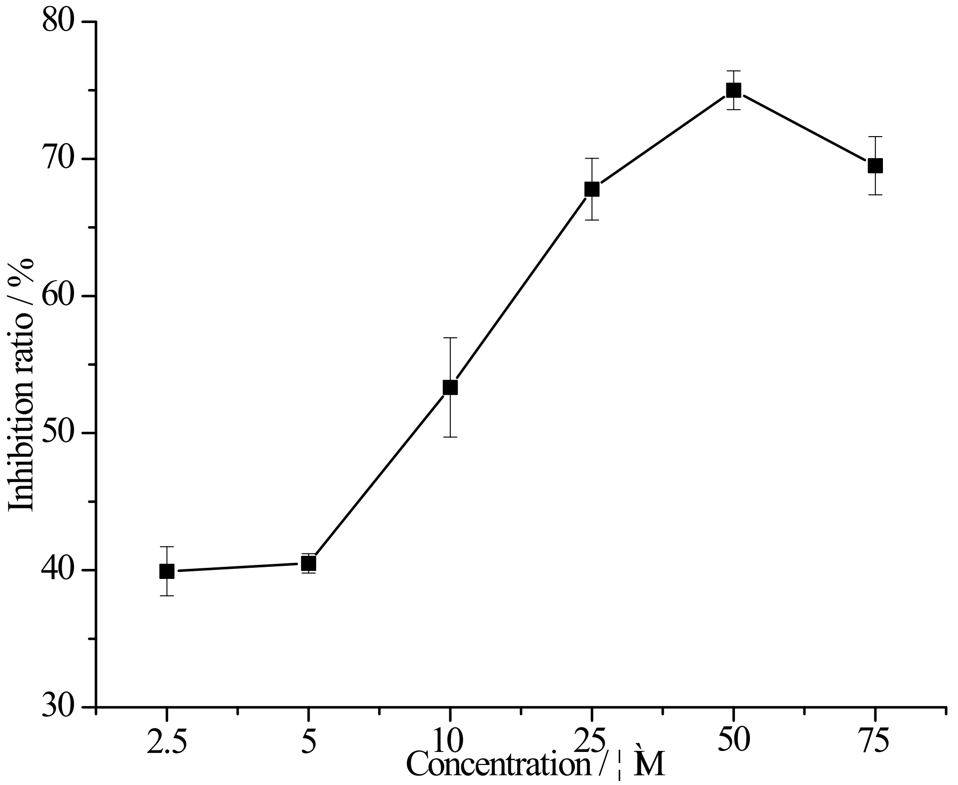

(3) The inhibitory activity of compound 3c on mTyr

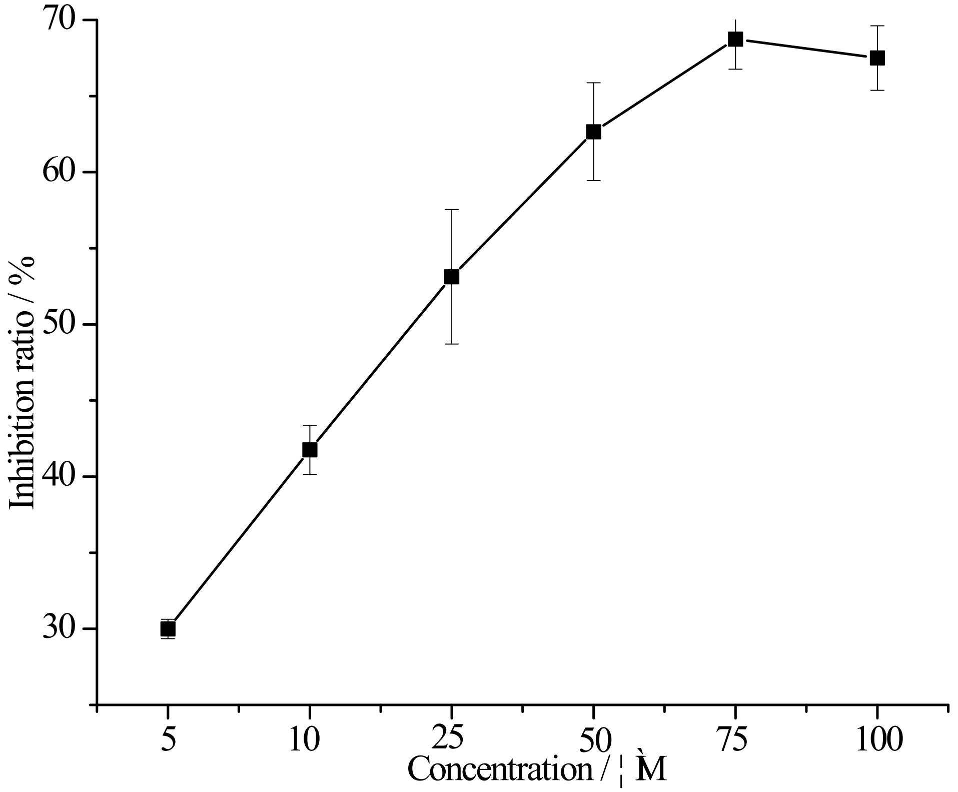

(4) The inhibitory activity of compound 3d on mTyr

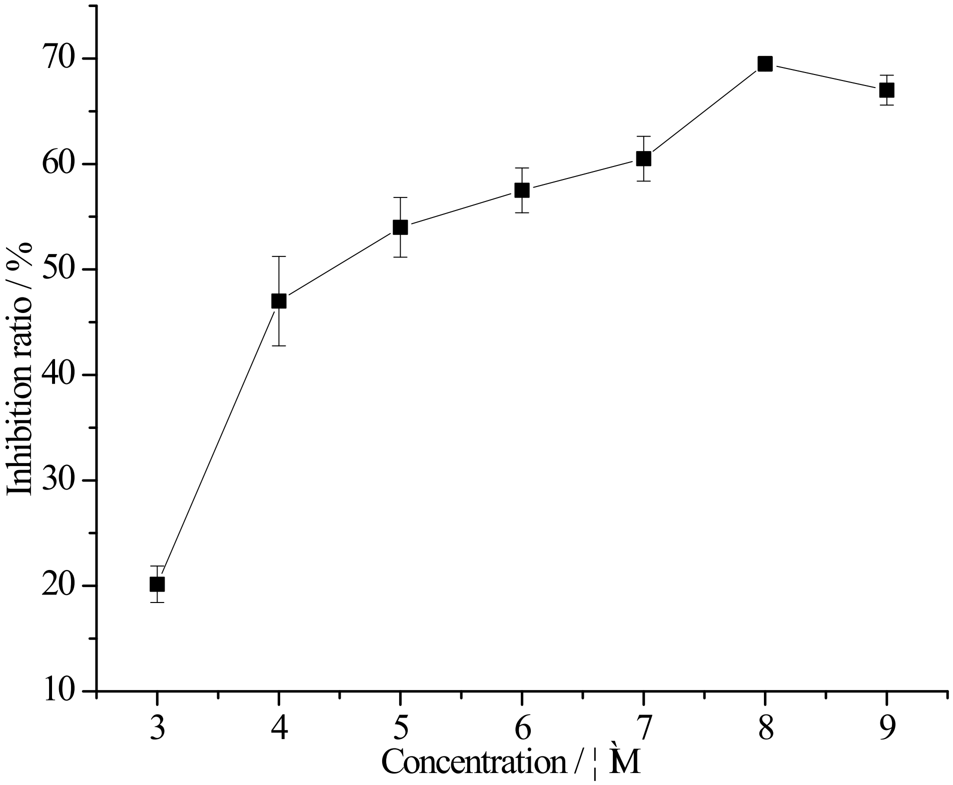

(5) The inhibitory activity of compound 3e on mTyr

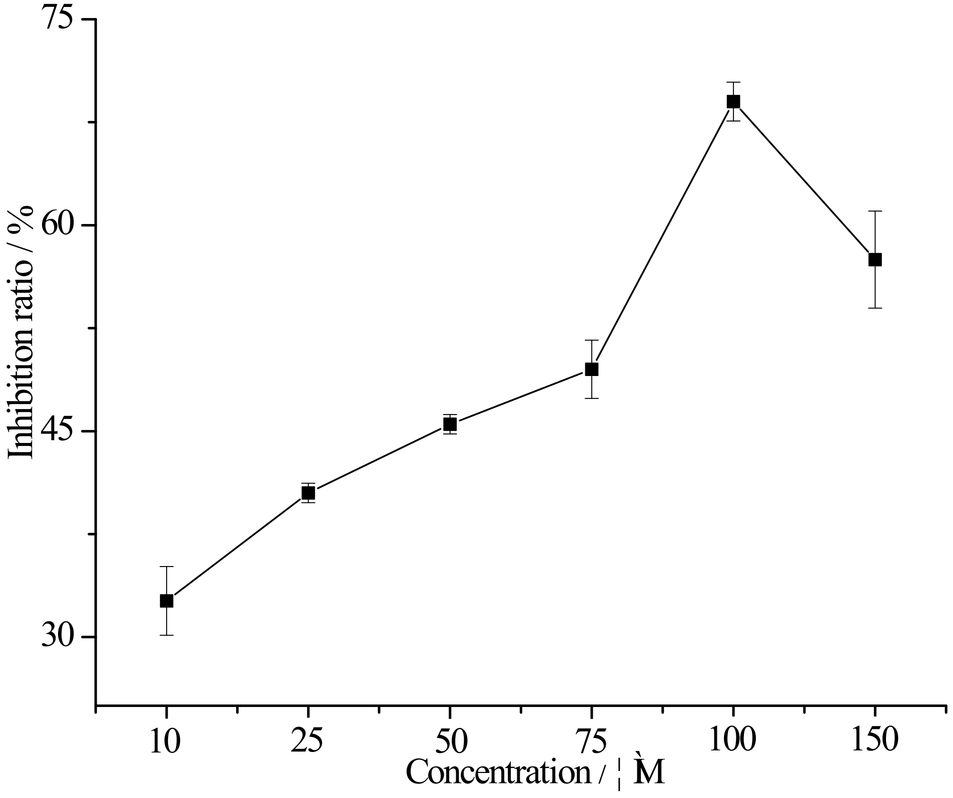

(6) The inhibitory activity of compound 3f on mTyr

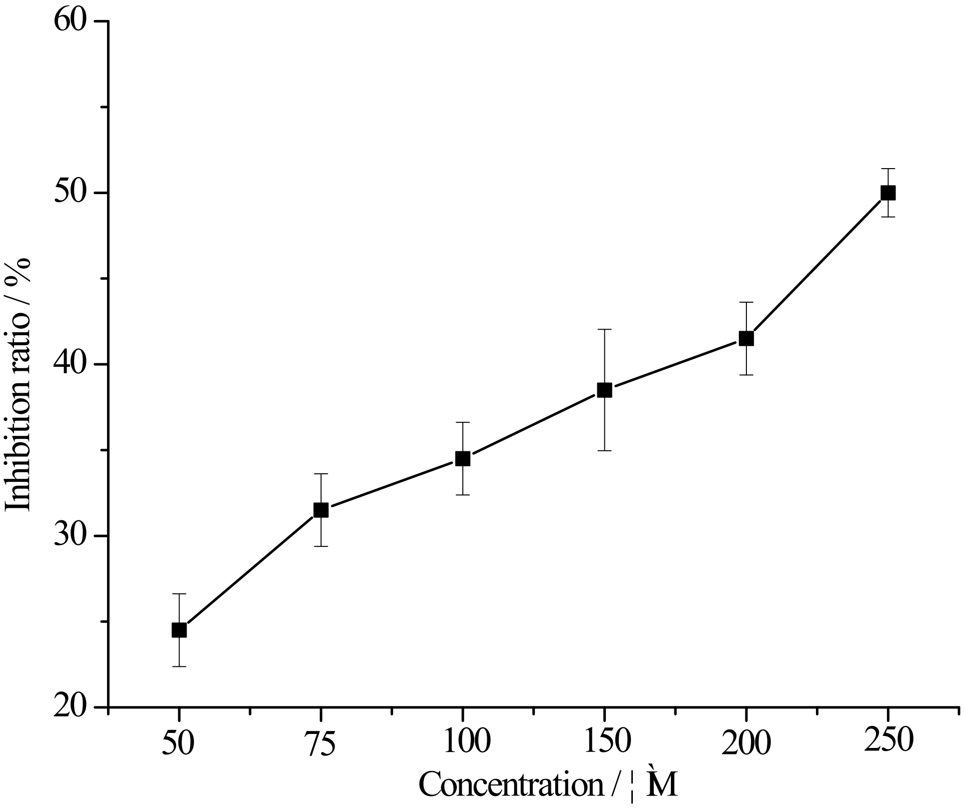

(7) The inhibitory activity of compound 3g on mTyr

Figure S3. The inhibitory activity of compounds 3a-3g on mTyr

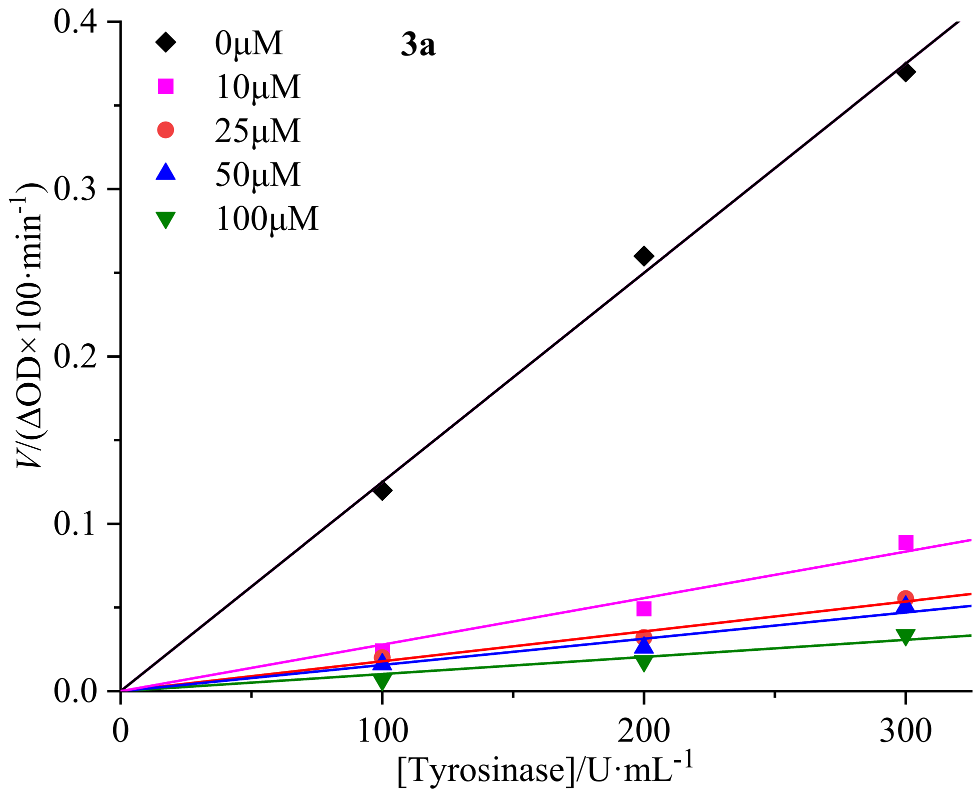

A

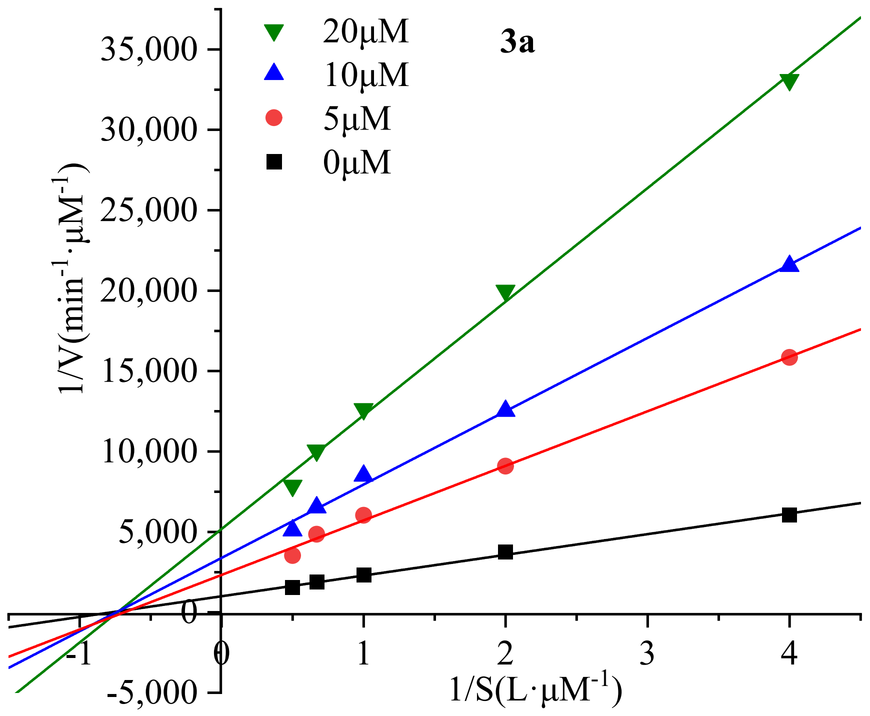

B

（1）Compound 3a

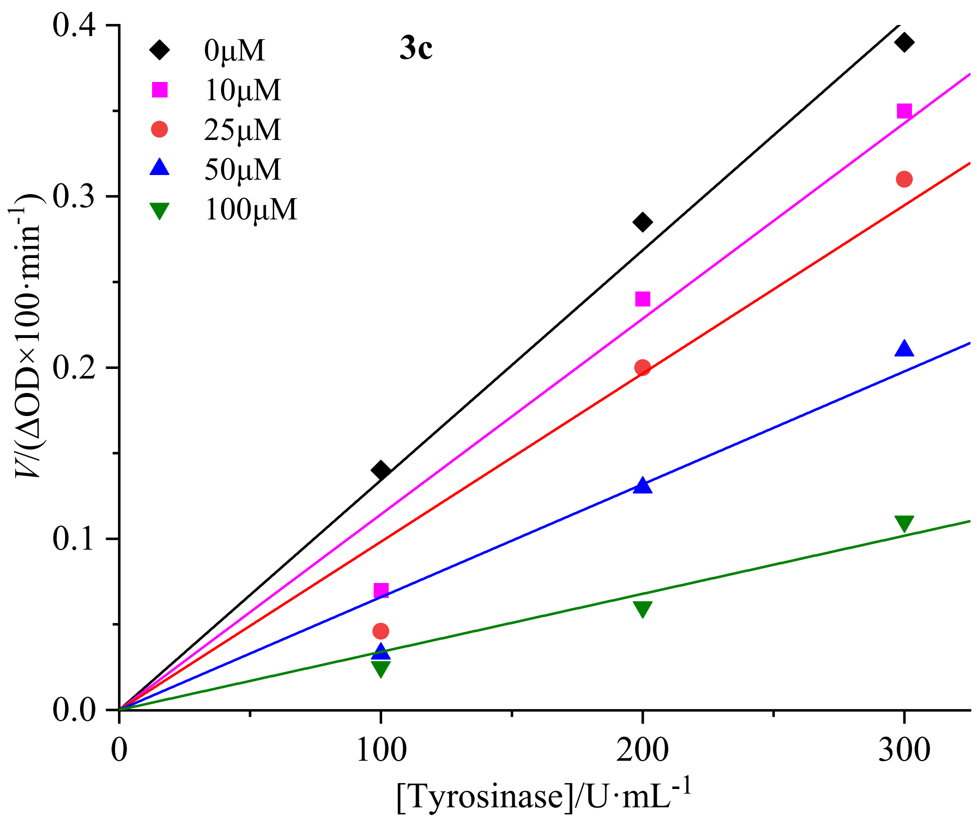

3c
0μM
10μM
25μM
50μM
100μM
V/(ΔOD×100·min-1)
[Tyrosinase]/U·mL-1
0.0
0.1
0.2
0.3
0.4
0
100
200
300

A

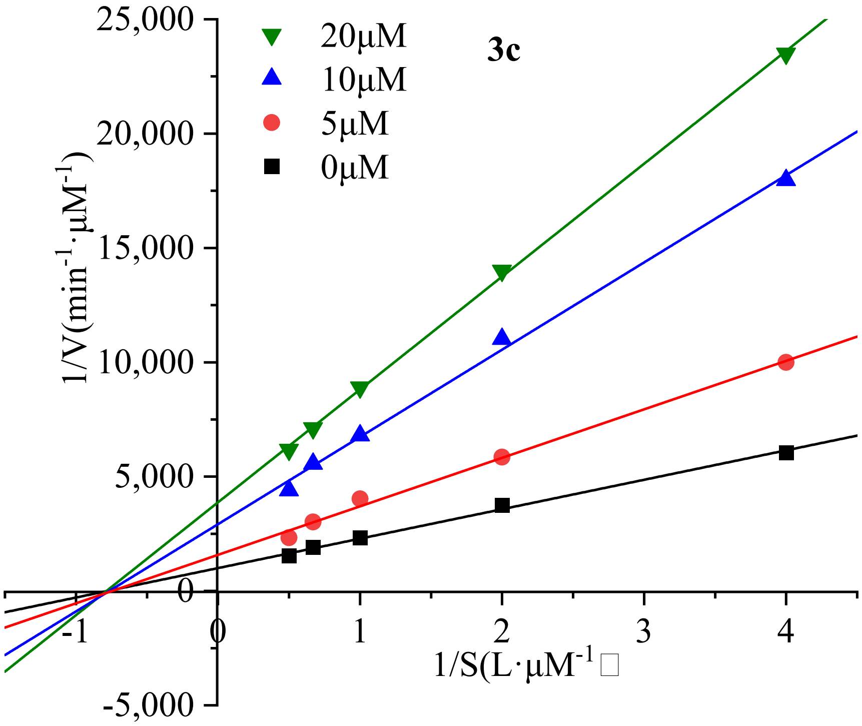

3c
20μM
10μM
5μM
0μM
1/V(min-1·μM-1)
1/S(L·μM-1
25,000
20,000
15,000
10,000
5,000
0
-5,000
-1
0
1
2
3
4

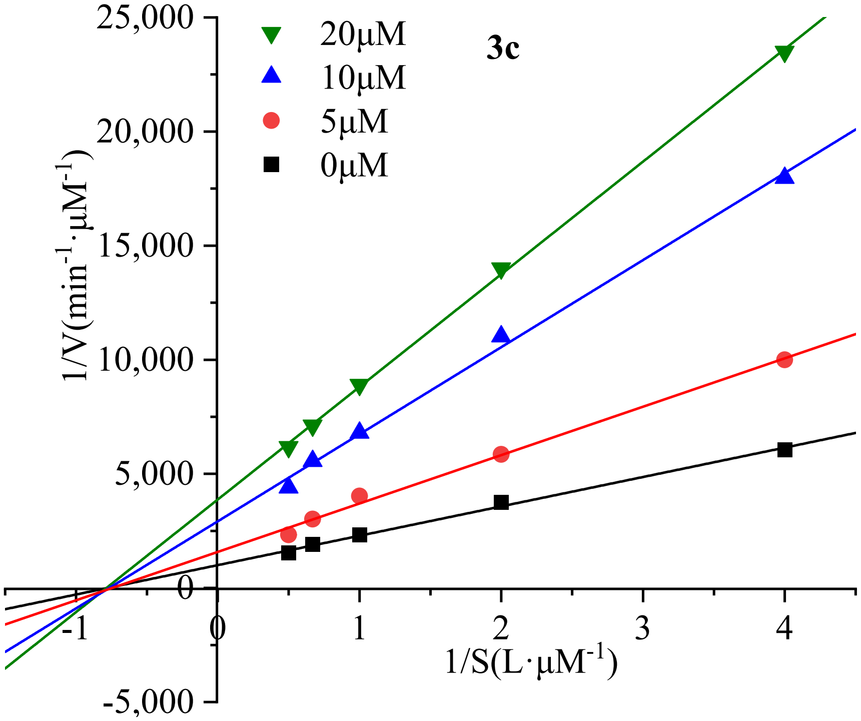

B

（2）Compound 3c

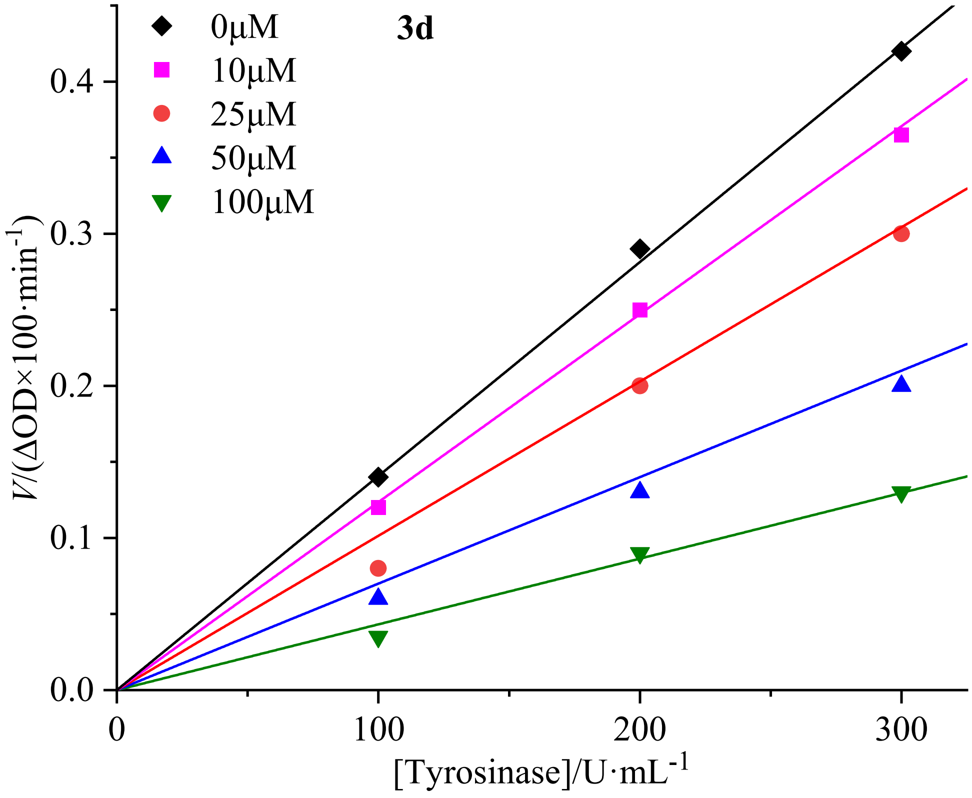

A

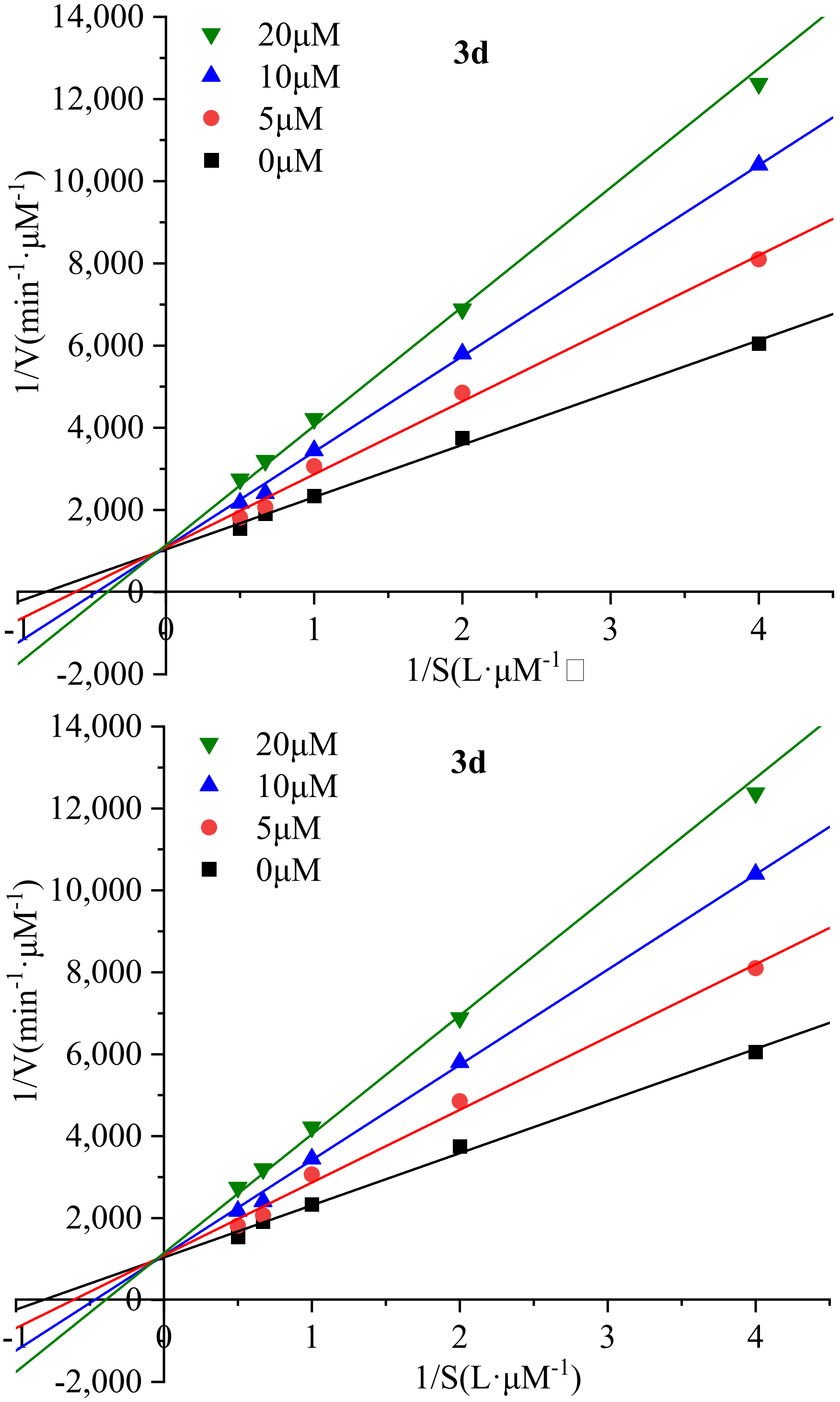

B

（3）Compound 3d

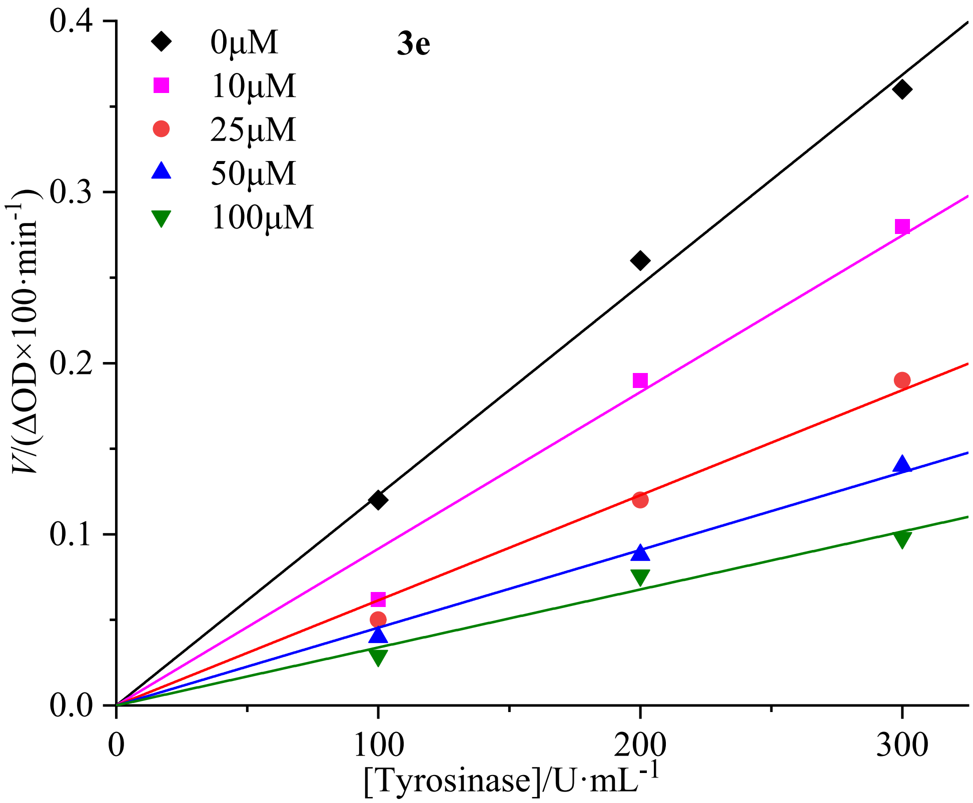
3e
0μM
10μM
25μM
50μM
100μM
V/(ΔOD×100·min-1)
0.0
0.1
0.2
0.3
0.4
0
100
200
300
[Tyrosinase]/U·mL-1

A

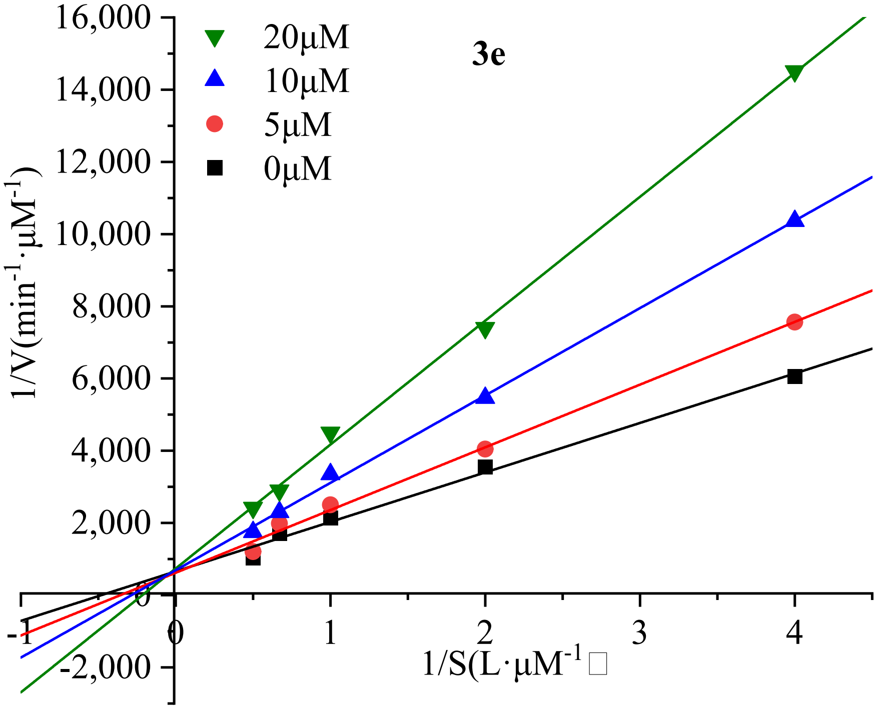
3e
20μM
10μM
5μM
0μM
1/V(min-1·μM-1)
16,000
14,000
12,000
10,000
8,000
6,000
4,000
2,000
0
-2,000
-1
0
1
2
3
4
1/S(L·μM-1)

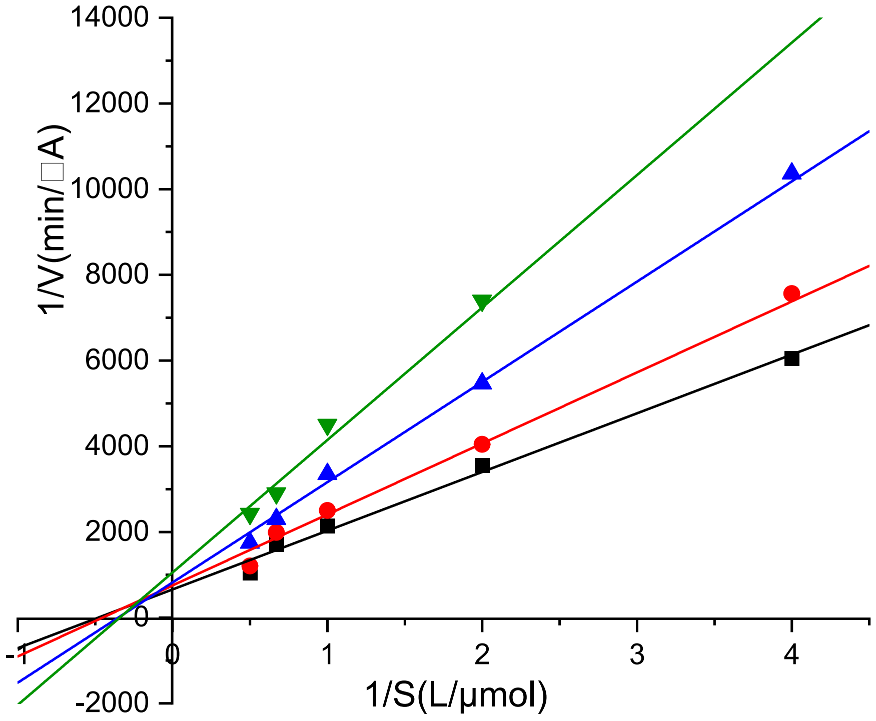

B

（4）Compound 3e

Figure S4 Inhibition reversibility (A) and inhibition type (B) of compound 3a, 3c-3e on mTyr.

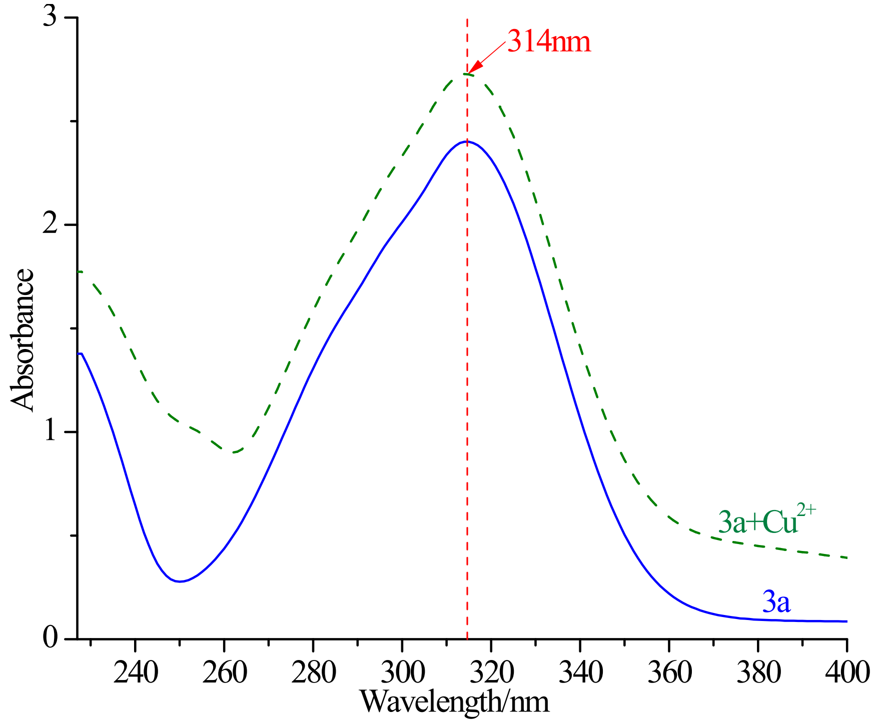

Compound 3a

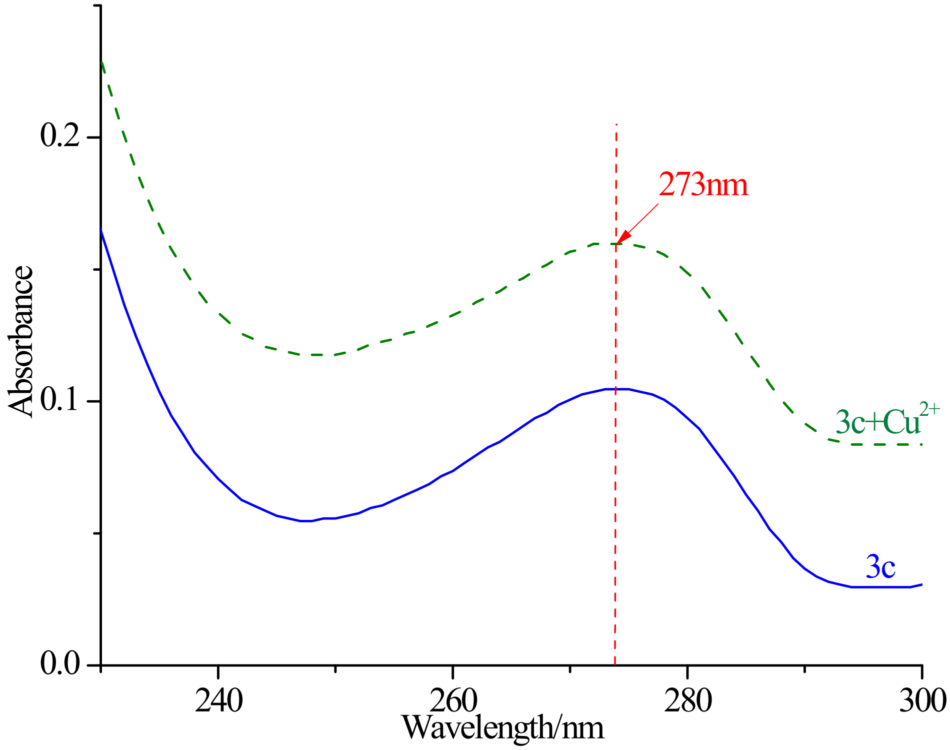

Compound 3c

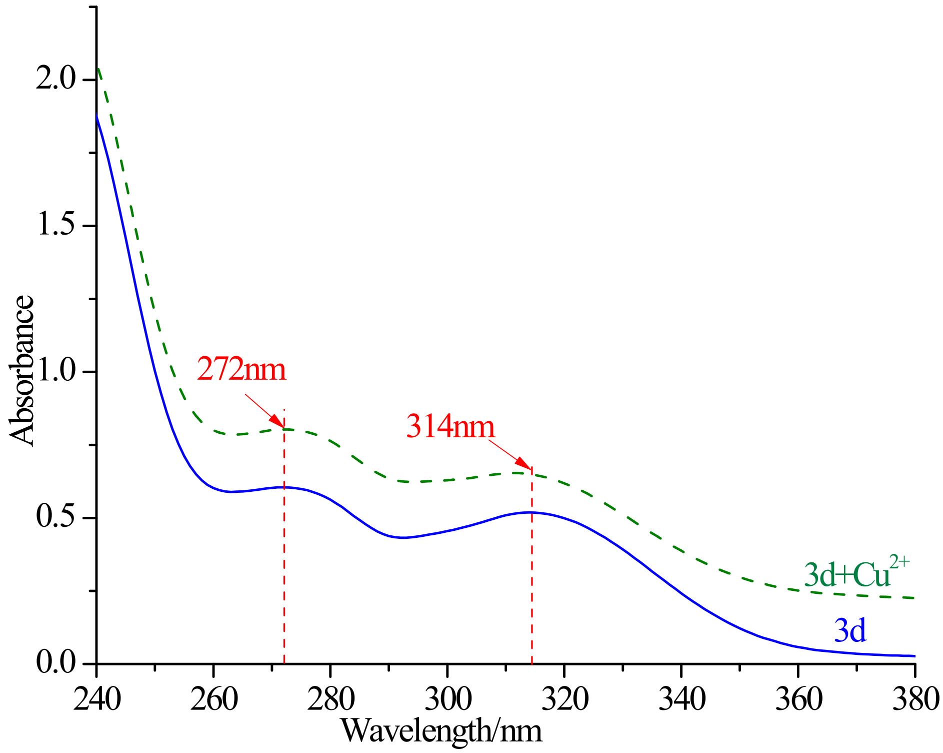

Compound 3d

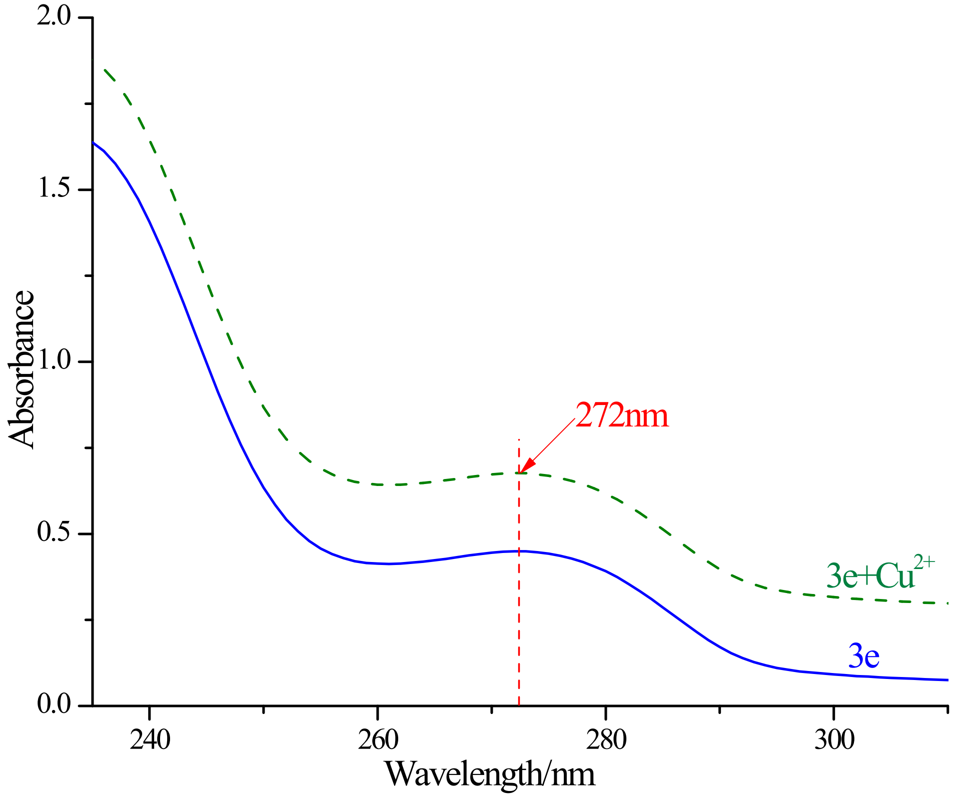

Compound 3e

Figure S5. UV Spectra of compound 3a, 3c-3e before and after interaction with mTyr

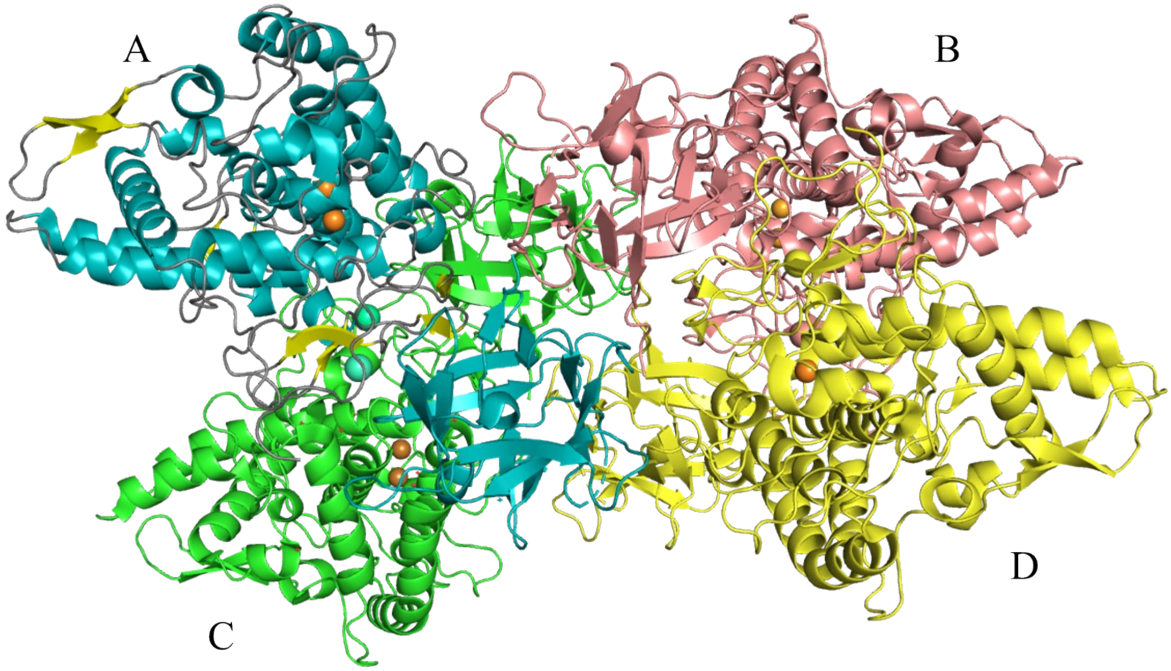

Figure S6. The crystal structure of mTyr (PDB ID: 2Y9X)

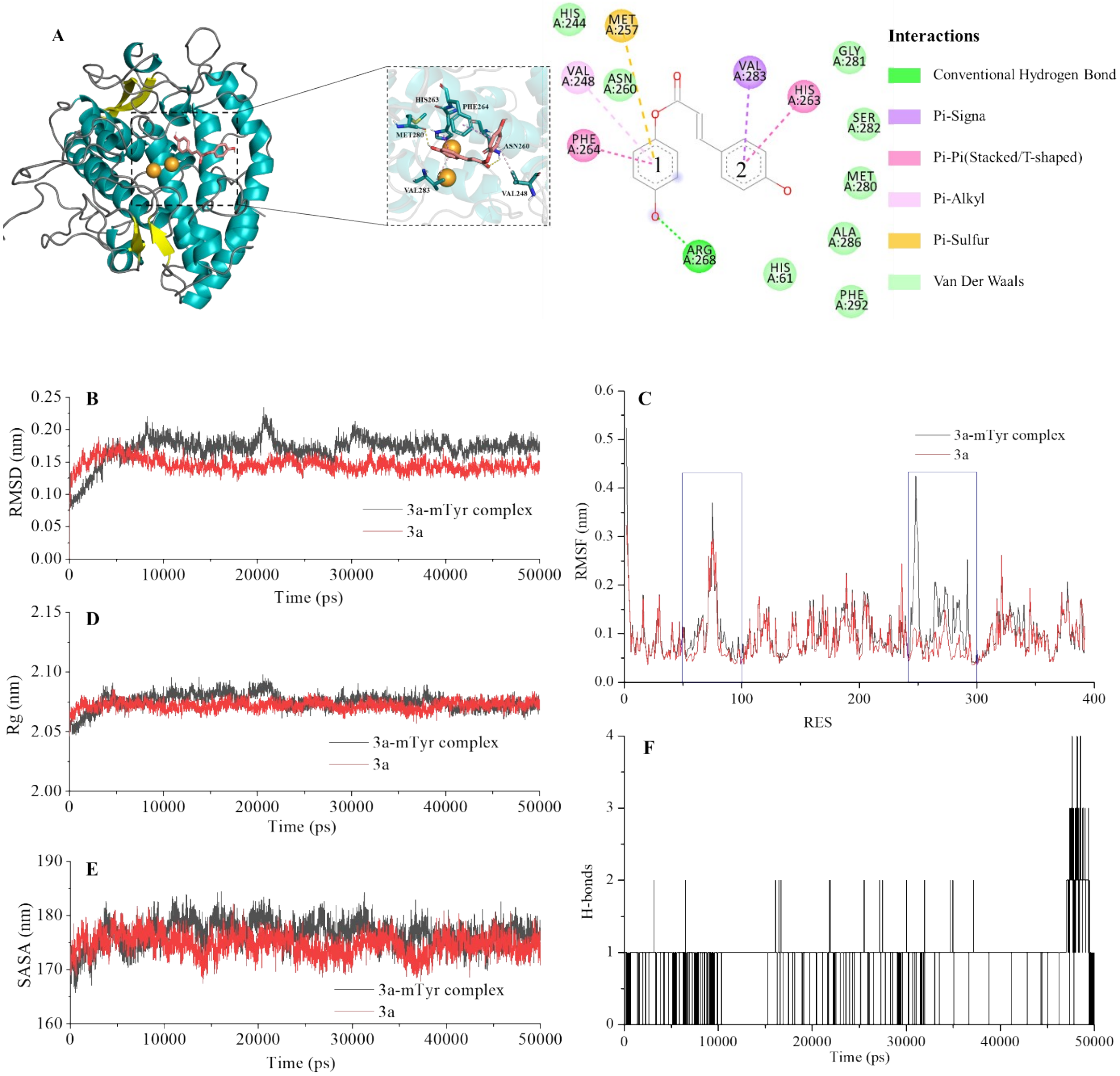
A
HIS263
PHE264
MET280
ASN260
VAL283
VAL248
HIS A:244
MET A:257
VAL A:248
ASN A:260
PHE A:264
VAL A:283
HIS A:263
GLY A:281
SER A:282
MET A:280
ALA A:286
ARG A:268
HIS A:61
PHE A:292
Interactions
Conventional Hydrogen Bond
Pi-Signa
Pi-Pi(Stacked/T-shaped)
Pi-Alkyl
Pi-Sulfur
Van Der Waals
B
RMSD (nm)
Time (ps)
3a-mTyr complex
3a
C
RMSF (nm)
RES
D
Rg (nm)
E
SASA (nm)
F
H-bonds

Compound 3a

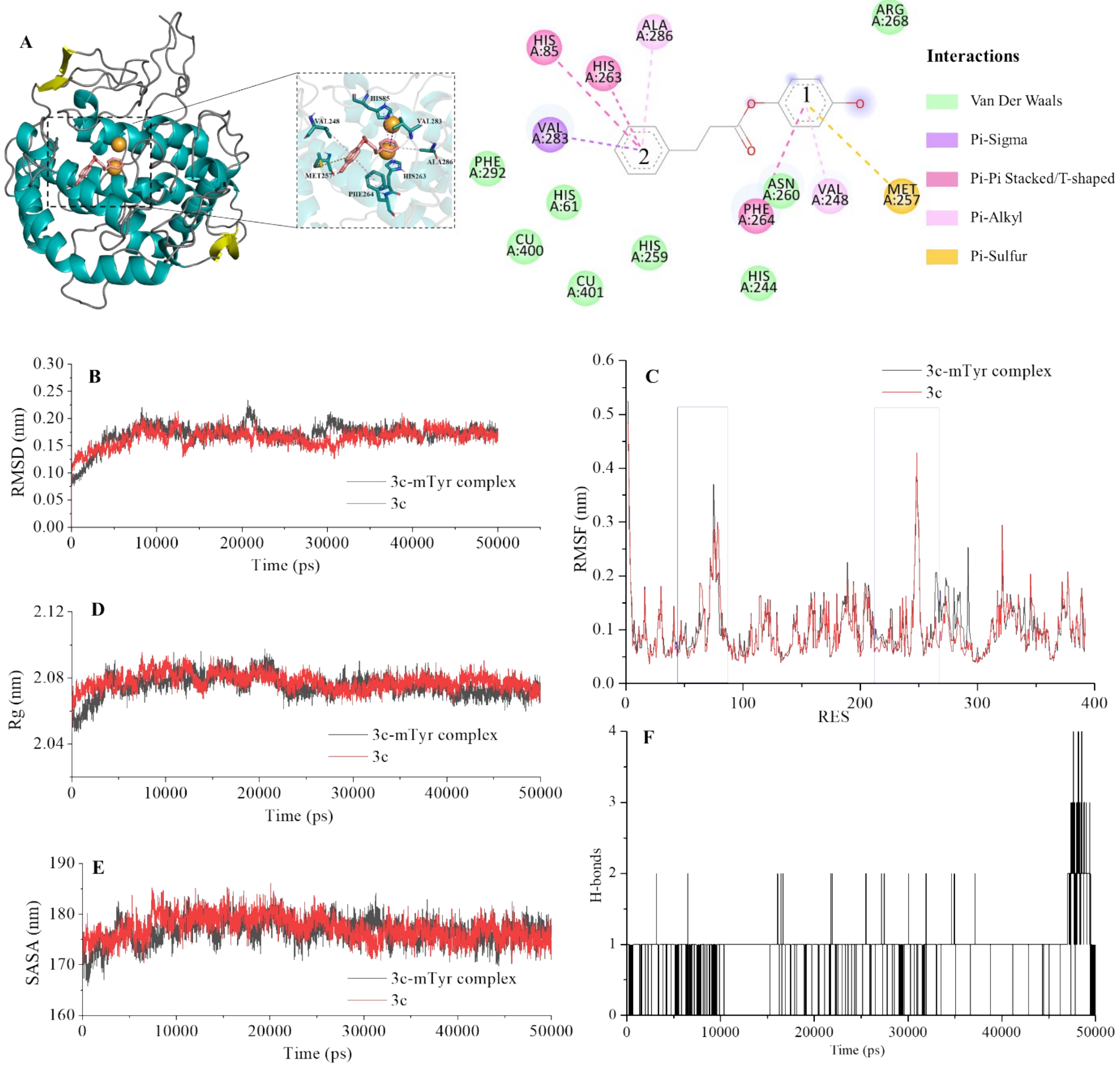
A
HIS85
VAL 248
VAL283
MET257
ALA286
HIS263
PHE264
HIS
A:85
HIS
A:263
ALA
A:286
ARG
A:268
VAL
A:283
PHE
A:292
HIS
A:61
CU
A:400
CU
A:401
HIS
A:259
ASN
A:260
PHE
A:264
VAL
A:248
MET
A:257
HIS
A:244
Interactions
Van Der Waals
Pi-Sigma
Pi-Pi Stacked/T-shaped
Pi-Alkyl
Pi-Sulfur
B
RMSD (nm)
Time (ps)
3c-mTyr complex
3c
C
RMSF (nm)
RES
3c-mTyr complex
3c
D
Rg (nm)
Time (ps)
3c-mTyr complex
3c
E
SASA (nm)
Time (ps)
3c-mTyr complex
3c
F
H-bonds
Time (ps)

Compound 3c

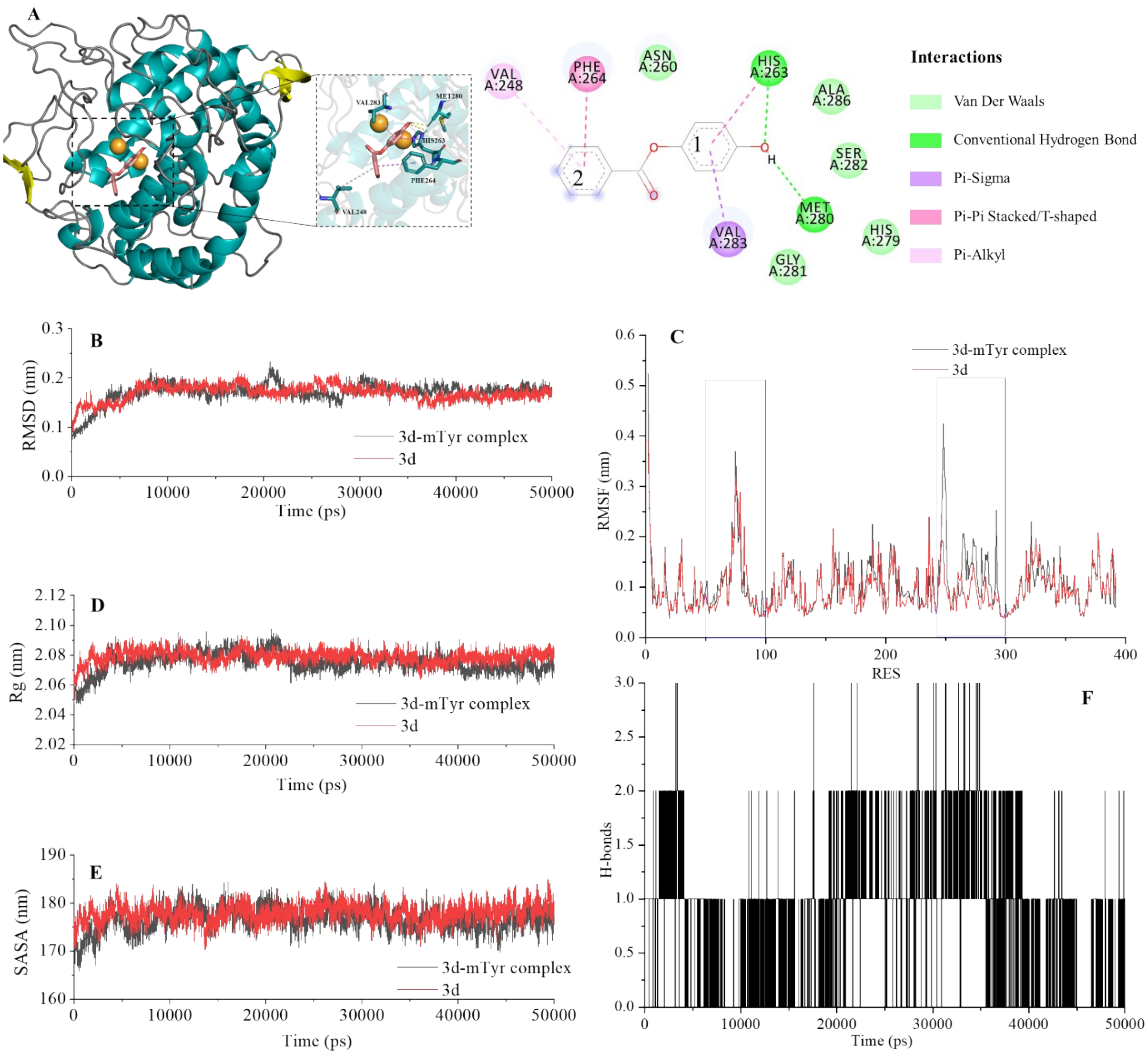
A
VAL283
MET280
HIS263
PHE264
VAL248
VAL A:248
PHE A:264
ASN A:260
HIS A:263
ALA A:286
SER A:282
MET A:280
HIS A:279
VAL A:283
GLY A:281
Interactions
Van Der Waals
Conventional Hydrogen Bond
Pi-Sigma
Pi-Pi Stacked/T-shaped
Pi-Alkyl
B
RMSD (nm)
Time (ps)
3d-mTyr complex
3d
C
RMSF (nm)
RES
D
Rg (nm)
E
SASA (nm)
F
H-bonds

Compound 3d

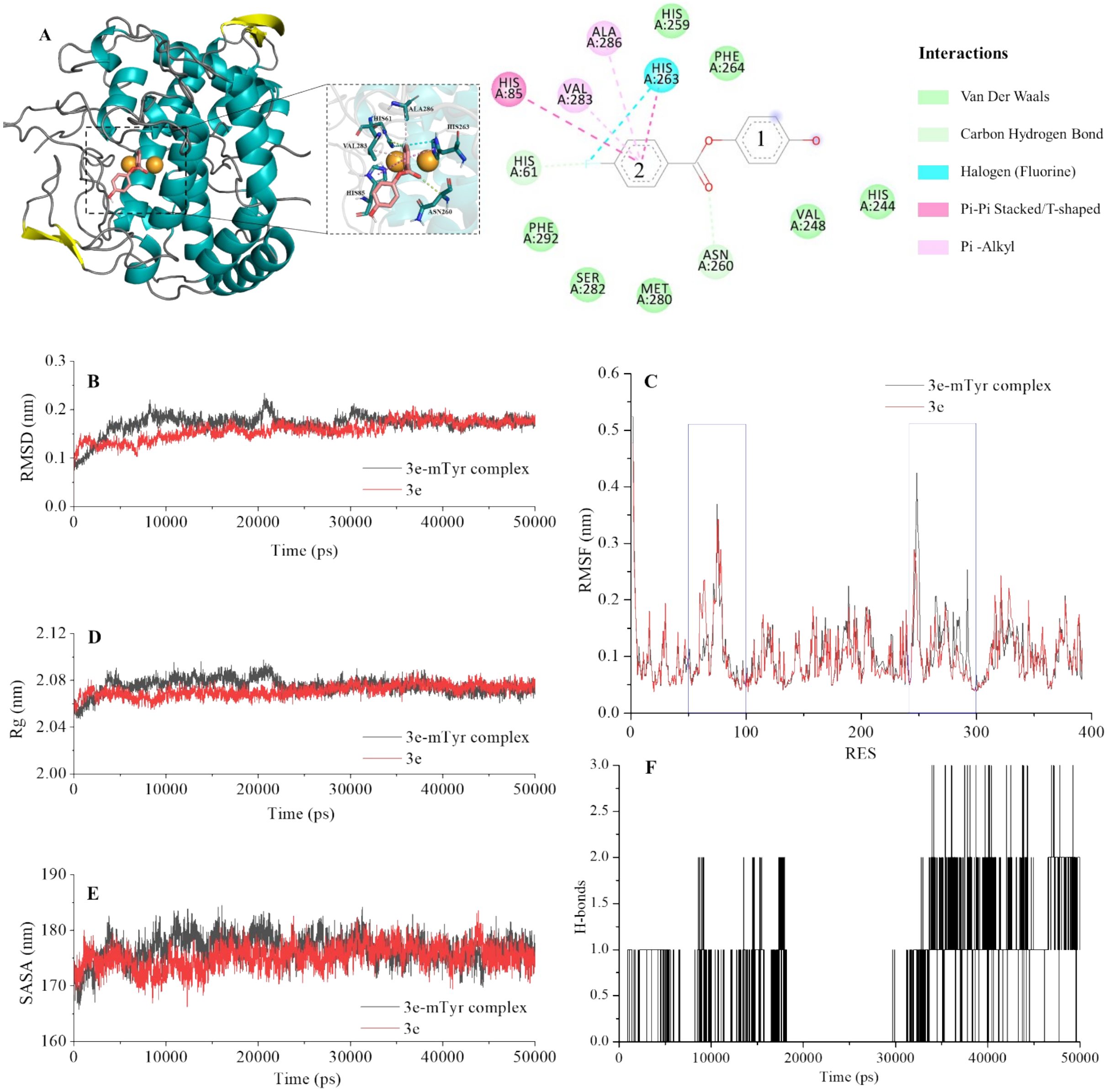

Compound 3e

Figure S7. Docking model for compound 3a, 3c-3e with mTyr (A) and Molecular dynamics results of compound 3a, 3c-3e and (3a, 3c-3e) -mTyr complex with: B. RMSD, C. RMSF, D. Rg, E. SASA, and F. H-bonds

Table S1. Linear fitting equation, Michaelis constant (K_m), maximum reaction rate (V_m), and inhibition type for mTyr at varying concentrations of compounds 3a-3e

Compound	Concentration (μM)	Linear fitting equation	R^2	K_m (μM)	V_m ($\times 10^3$, min^{-1})	Inhibition type
3a	0	Y=1000.25+1287.41X	0.9991	1.2871	0.9998	Noncompetitive
	5	Y=2340.91+3012.40X	0.9958	1.2870	0.4272	
	10	Y=3516.19+4525.44X	0.9963	1.2867	0.2843	
	20	Y=5446.87+7064.94X	0.9965	1.2869	0.1836	
3b	0	Y=333.72+1645.85X	0.9902	4.9318	2.9965	Mix-noncompetitive
	1	Y=1320.62+2456.41X	0.9949	2.6173	0.7572	
	2	Y=2889.49+3833.45X	0.9964	1.3267	0.3461	
	4	Y=4662.26+5183.79X	0.9982	1.1119	0.2145	
3c	0	Y=1000.25+1287.41X	0.9991	1.2871	0.9998	Noncompetitive
	5	Y=1580.45+2034.21X	0.9990	1.2870	0.6327	
	10	Y=2920.18+3759.70X	0.9991	1.2873	0.3424	
	20	Y=3875.12+4950.54X	0.9999	1.2774	0.2581	
3d	0	Y=1084.97+1272.89X	0.9961	1.1732	0.9217	Competitive
	5	Y=1085.12+1776.46X	0.9985	1.6371	0.9216	
	10	Y=1086.06+2324.89X	0.9996	2.1407	0.9208	
	20	Y=1085.95+2900.15X	0.9967	2.6706	0.9209	
3e	0	Y=679.35+1370.31X	0.9904	2.0171	1.472	Competitive
	5	Y=678.55+1736.97X	0.9943	2.5598	1.474	
	10	Y=680.49+2419.72X	0.9983	3.5558	1.470	
	20	Y=681.12+3431.14X	0.9983	5.0375	1.468	

Table S2 Docking energy and bonding condition of compounds 3a-3e with mTyr

Compounds	Docking energy (kcal·mol^{-1})	Bonding condition			
		Hydrogen Bonds	Hydrophobic	Miscellaneous (Pi-Sulfur)	Halogen (Fluorine)
3a	-7.6	Ph1O←Arg 268 (6.66 Å, Conventional Hydrogen Bond)	Ph1←Phe264 (6.68Å, Pi-Pi Stacked/T-shaped); Ph1←Val248 (5.16Å, Pi-alkyl); Ph2←His263 (5.57Å, Pi-Pi Stacked/T-shaped); Ph2←Val283 (5.11Å, Pi-Sigma).	Ph1←Met257 (6.50 Å)	
3b	-7.2	Ph1OH←Met280(5.01 Å, Conventional Hydrogen Bond)	Ph1←His263 (5.32Å, Pi-Pi Stacked); Ph1←Val283 (4.89Å, Pi- Sigma); Ph2←Val248 (5.31 Å, Pi-Sigma).	Ph2←Met257 (6.57 Å)	
3c	-6.7		Ph1←Val248 (5.57Å, Pi-Alkyl); Ph1←Phe264 (6.74Å, Pi-Pi Stacked/T-shaped); Ph2←Val283 (4.26Å, Pi-Sigma); Ph2←His85/His263 (5.47/5.48Å, Pi-Pi Stacked/T-shaped); Ph2←Ala 286 (6.53Å, Pi-Alkyl).	Ph1←Met257(7.58 Å)	
3d	-7.0	Ph1OH←Met280(4.88 Å, Conventional)Hydrogen Bond; Ph1O←His 263 (4.06 Å, Conventional Hydrogen Bond)	Ph1←His 263 (5.52Å, Pi-Pi Stacked/T-shaped); Ph1←Val283 (4.82Å, Pi-Sigma); Ph2←Val248 (6.29Å, Pi-Alkyl); Ph2←Phe264 (6.62Å, Pi-Pi Stacked/T-shaped).		
3e	-6.7	Ph2F←His61 (5.50 Å, Carbon Hydrogen Bond)	Ph2←Val283 (4.42Å, Pi-Signa); Ph2← Val283/Ala286 (4.63/6.95Å, Pi-Alkyl); Ph2←His85/His263 (7.42/4.57Å, Pi-Pi Stacked/T-shaped).		Ph2F←His 263 (5.41 Å Halogen (Fluorine))
